# Extreme red shifted SERS nanotags†
†Electronic supplementary information (ESI) available: General experimental details for the synthesis and characterization of dyes **1–14**, intermediates **15–17**, and HGNs; general description of the chemometrics used for principal component analysis; figures of SERS spectra of dye/HGN nanotags with 1280 nm excitation; extinction spectrum for HGNs; SERS spectra of Au and Ag nanoparticles and dye **13** aggregated with KCl; SERS spectra of dyes **13** and **14** on HGN not aggregated with KCl; details of mean plane angle calculations, tables of crystallographic data, atomic coordinates and equivalent isotropic displacement parameters, anisotropic placement parameters, bond lengths, bond angles, and hydrogen atom coordinates and isotropic displacement parameters for dye **14**. CCDC 1040064. For ESI and crystallographic data in CIF or other electronic format see DOI: 10.1039/c4sc03917c



**DOI:** 10.1039/c4sc03917c

**Published:** 2015-01-21

**Authors:** Matthew A. Bedics, Hayleigh Kearns, Jordan M. Cox, Sam Mabbott, Fatima Ali, Neil C. Shand, Karen Faulds, Jason B. Benedict, Duncan Graham, Michael R. Detty

**Affiliations:** a Department of Chemistry , University at Buffalo , The State University of New York , New York 14260 , United States . Email: mdetty@buffalo.edu; b Centre for Molecular Nanometrology , WestCHEM , Department of Pure and Applied Chemistry , University of Strathclyde , 295 Cathedral Street , Glasgow , G1 1XL , United Kingdom . Email: duncan.graham@strath.ac.uk; c Dstl , Porton Down , Salisbury , UK

## Abstract

Surfaced enhanced Raman scattering (SERS) nanotags operating with 1280 nm excitation were constructed from reporter molecules selected from a library of 14 chalcogenopyrylium dyes containing phenyl, 2-thienyl, and 2-selenophenyl substituents and a surface of hollow gold nanoshells (HGNs). These 1280 SERS nanotags are unique as they have multiple chalcogen atoms available which allow them to adsorb strongly onto the gold surface of the HGN thus producing exceptional SERS signals at this long excitation wavelength. Picomolar limits of detection (LOD) were observed and individual reporters of the library were identified by principal component analysis and classified according to their unique structure and SERS spectra.

## Abstract 2

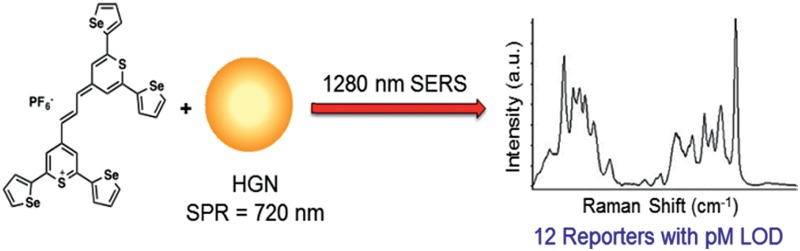
Extreme red-shifted nanotags have been developed and they provide effective SERS with picomolar detection limits when excited at 1280 nm.

## Introduction

Surface-enhanced Raman scattering (SERS) has been utilized as an extremely sensitive analytical tool in the study of biological systems. The combination of a metallic nanoparticle and an organic dye reporter molecule provide SERS nanotags that can be used to detect target molecules using laser Raman spectroscopy or SERS microscopy.^1,2^ SERS nanotags^1,3^ and SERS reporters have been designed and utilized with the 785 nm laser^4,5^ for most medical applications. Although the optical absorptance of human tissue is minimal in the 600–800 nm window, the depth of penetration of infrared light increases at longer wavelengths due to decreased scattering, reaching a minimum near 1300 nm.^6^ The superior penetration depth of 1300 nm light *vs.* 800 nm light is documented,^7,8^ but Raman scattering at 1300 nm is so weak that it is impossible to use. Therefore, to exploit the advantages of the unique vibrational signatures produced by Raman scattering, surface enhancement of the signal must be used to operate at this longer wavelength of excitation.

SERS nanotags operating at 1064 nm have been described using crystal violet, rhodamine 6G, methylene blue, and 9-aminoacridine as reporter molecules.^9,10^ A direct comparison of the 1064 nm (Ti:sapphire) and 1280 nm (Cr:forsterite) lasers on tissue samples (no SERS) showed that the 1280 nm laser excitation gave reduced sample burning, limited photo-bleaching, reduced background fluorescence/autofluorescence, and greater penetration depth into biological tissues.^6,11^ The superior penetration of 1280 nm light in turbid media such as tissue and blood^7,9^ has been utilized in both optical coherence tomography^7,12^ and fluorescence microscopy.^13^ To date, there appear to be no SERS nanotags compatible with a 1280 nm excitation laser and a great need remains for effective SERS nanotags operating with 1280 nm excitation.

**Fig. 1:**
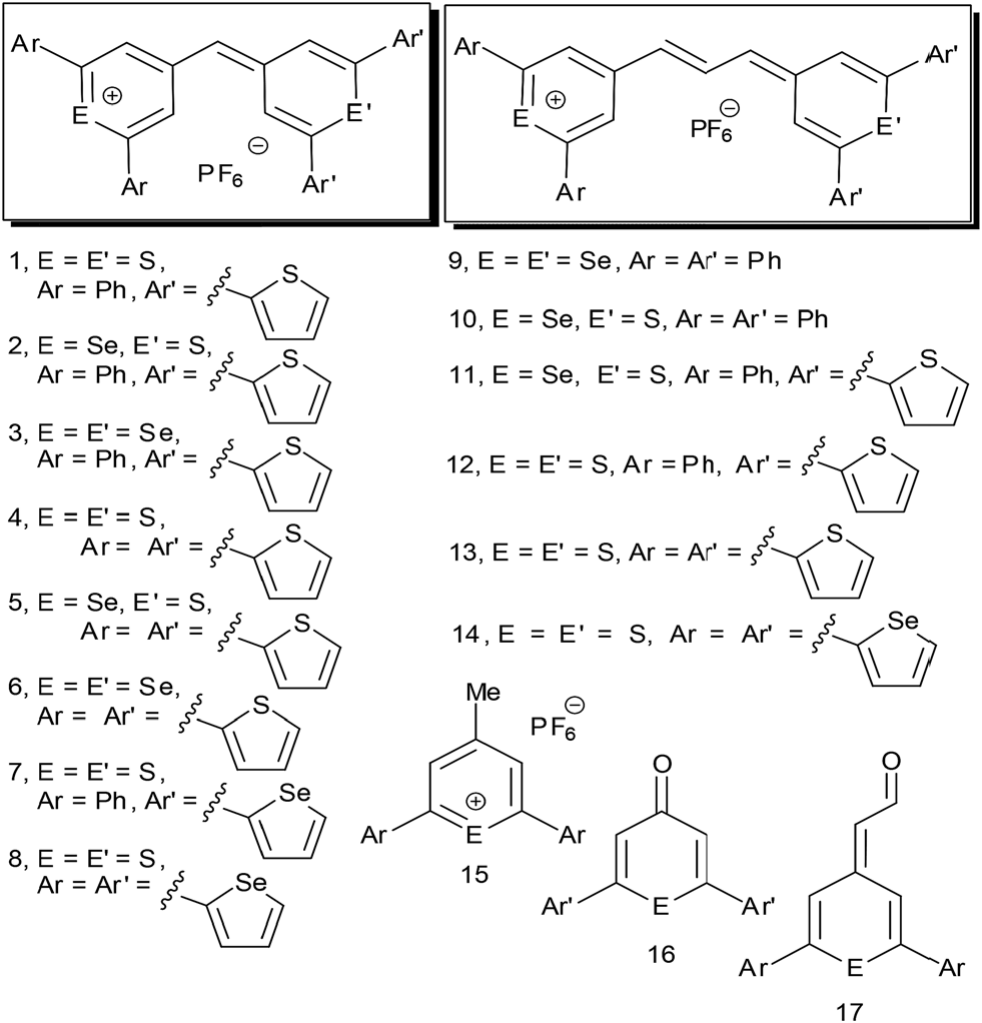
Chalcogenopyrylium monomethine (**1–8**) and trimethine dyes (**9–14**) from building blocks **15–17**.

We report the design of 1280 nm SERS nanotags based on hollow gold nanoshells (HGNs)^14^ and reporter molecules selected from a small library of (chalcogenopyranyl)chalcogenopyrylium monomethine (**1–8**) and trimethine dyes (**9–14**, Fig. 1). These dyes are substituted with phenyl, 2-thienyl, and 2-selenophenyl substituents at the 2- and 6-positions of the pyrylium/pyranyl rings, which allow them to bind strongly to the HGN surface with multiple attachment groups. Dye **14** with two sulfur atoms in the thiopyrylium/thiopyranyl core and four 2-selenophenyl substituents at the 2,2′,6,6′-positions was exceptionally bright in this library of reporters with 1280 nm excitation. All fourteen members of the reporter library can be uniquely identified by principal component analysis of their SERS spectra.

## Results and discussion

### Reporter library synthesis and properties

The library of dyes **1–14** was constructed by condensation of 4-methylthiopyrylium and 4-methylselenopyrylium salts **15** either with chalcogenopyranones **16** or with (4-chalcogeno-pyranylidene)acetaldehyde derivatives **17** in acetic anhydride to give monomethine dyes **1–8** or trimethine dyes **9–14**, respectively (Fig. 1).^15^ 4-Methylthiopyrylium and 4-methylseleno-pyrylium salts **15** were prepared by the addition of MeMgBr to the corresponding chalcogenopyranone **16** followed by treatment with aqueous HPF_6_. Synthetic details are provided in the ESI.† Values of absorption maxima, *λ*_max_, in CH_2_Cl_2_ for **1–8** varied from 653 nm for **1** to 724 nm for **6** and values of the molar extinction coefficient, *ε*, were in the range of 1.1 × 10^5^ to 1.5 × 10^5^ M^–1^ cm^–1^ (Table 1). For trimethine dyes **9–14**, values of *λ*_max_ in CH_2_Cl_2_ varied from 784 nm for dye **10** to 826 nm for dye **14** while values of *ε* were in the range of 2.0 × 10^5^ to 2.8 × 10^5^ M^–1^ cm^–1^ (Table 1). The interchange of S and Se atoms in the chalcogenopyrylium backbone, the use of monomethine and trimethine bridges, and the interchange of phenyl, 2-thienyl, and 2-selenophenyl substituents at the 2-,2′-, 6- and 6′-positions allow the fine tuning of wavelengths of absorption^15^ and allow each dye to have a unique Raman fingerprint.

**Table 1:** Values of the absorption maximum (*λ*_max_), molar extinction coefficient (*ε*) and calculated LOD values from the SERS experiment with associated standard deviation (s.d.) error for chalcogenopyrylium dyes **1–14**

Dye	*λ*_max_, nm (CH_2_Cl_2_)	*ε*, M^–1^ cm^–1^ (CH_2_Cl_2_)	LOD, pM^*a*^
**1**	653	1.3 × 10^5^	21.8 ± 1.9
**2**	676	1.3 × 10^5^	29.4 ± 2.7
**3**	699	1.5 × 10^5^	32.8 ± 2.4
**4**	676	1.2 × 10 ^5^	—
**5**	698	1.1 × 10^5^	5.4 ± 0.6
**6**	724	1.3 × 10^5^	—
**7**	659	1.4 × 10^5^	4.6 ± 0.5
**8**	687	1.1 × 10^5^	3.4 ± 0.2
**9**	806	2.5 × 10^5^	9.1 ± 0.7
**10**	784	2.0 × 10^5^	5.9 ± 0.3
**11**	810	2.5 × 10^5^	1.8 ± 0.2
**12**	789	2.2 × 10^5^	6.6 ± 0.6
**13**	813	2.8 × 10^5^	1.5 ± 0.1
**14**	826	2.3 × 10^5^	1.5 ± 0.1

^*a*^SERS – LOD ± s.d.

### SERS spectra and SERS nanotags with dyes **1–14**

**Fig. 2:**
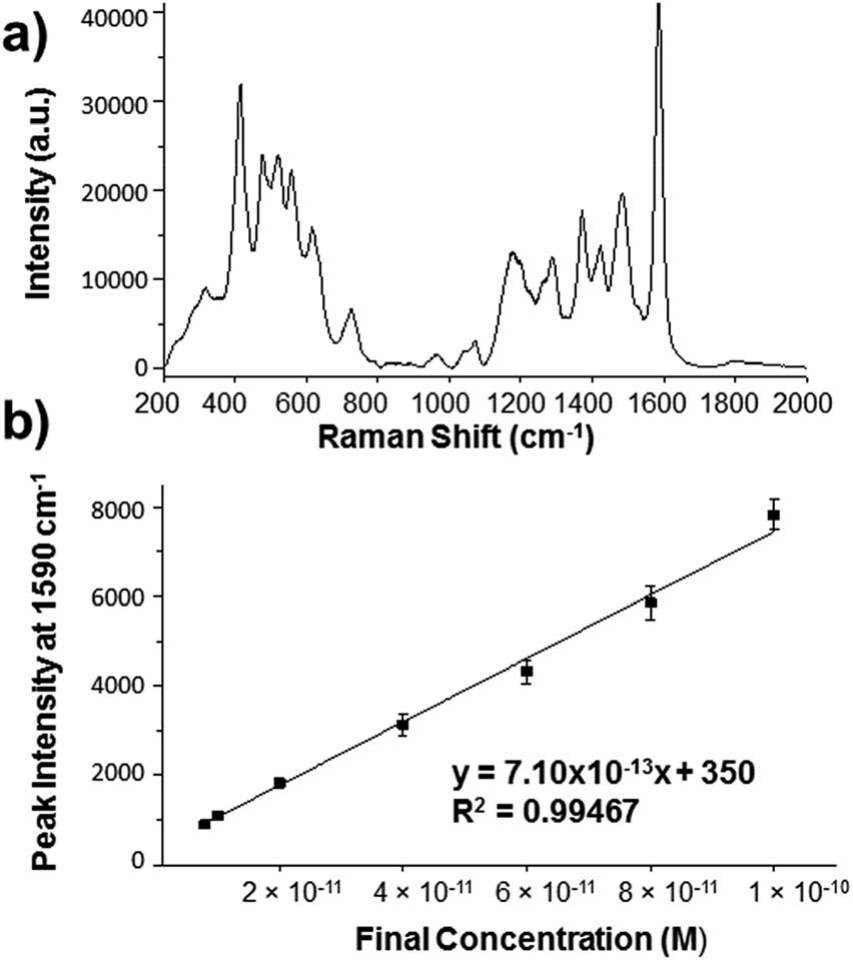
(a) SERS spectrum of dye **14** (10 μM) analyzed with HGNs (SPR recorded at 720 nm) and KCl (30 mM). The spectrum has been background corrected. (b) SERS particle dilution study for dye **14** with HGNs and KCl over the concentration range 1.93 nM to 6 pM. The limit of detection was calculated to be 1.5 ± 0.1 pM. Peak height at 1590 cm^–1^ was analysed by subtracting background ‘HGN only’ signal from each data point. Error bars represent one standard deviation resulting from 3 replicate samples and 5 scans of each. 1280 nm laser excitation was used for both (a) and (b) and an exposure time of 3 s for (a) and 7 s for (b) were employed in this analysis.

Raman scattering tends to be weak in the near infrared (NIR) region due to its dependence on the 4th power of the excitation frequency, but can be significantly enhanced by trapping molecules close to the gold surface of HGNs.^14^ The enhancement obtained from SERS is related to the frequency of the surface plasmon excited on the metal rather than the 4th power law.^16^ Therefore, to make SERS a viable method at 1280 nm, the surface plasmon resonance (SPR) should have some resonance with the NIR excitation source. The SERS spectrum of dye **14** is shown in Fig. 2a with the SERS spectra of dyes **1–13** in Fig. S1 of the ESI.† The HGNs have a SPR at 720 nm as shown in the extinction spectrum in Fig. S2 of the ESI.† Moreover, the extinction spectra showing that the SPR does not shift upon functionalizing HGNs with these dyes or with the addition of KCl, can also be seen in Fig. S2.† It is important to note that the plasmon resonance frequencies obtained do not match the excitation frequency at 1280 nm, however, it has been reported previously that for effective SERS to be achieved, the excitation wavelength does not need to match the wavelength of the plasmon maximum.^17^

The 1280 nm SERS nanotags, consist of three important components, the first being the SERS substrate. In addition to strong SERS properties,^18^ HGNs have small size (usually 50–80 nm), spherical shape and a strong tunable plasmon band from the visible to the NIR region.^19^ Commonly, Ag and Au spherical nanoparticles with plasmon bands in the visible region are used as SERS substrates.^20^ However, dyes **1–14** on these nanoparticles produced significantly weaker SERS signals than those observed with the HGNs (see Fig. S3, ESI†) due to their lack of red-shifted SPR.

The second necessary component of SERS nanotags is the Raman reporter. Since the SERS effect decreases exponentially as a function of distance from the nanoparticle,^21^ the Raman reporter must be near the gold surface. Dyes **1–14** all incorporate S and Se atoms in the chalcogenopyrylium core to provide attachment to the Au surface of the HGNs, while the 2-thienyl and 2-selenophenyl groups on select members of this library provide additional attachment points to gold. Earlier studies have shown that thiophenes^22^ and selenophenes^23^ are both capable of forming self-assembled monolayers on gold. Selenolates have also been shown to have greater affinity for gold than thiolates.^24^

As shown in Fig. 1 and in Fig. S1 (ESI†), dyes **1–14**, which are highly aromatic, produce vibrationally rich and intense SERS spectra with a laser excitation of 1280 nm. The trimethine dyes **9–14** produce more intense signals than their monomethine counterparts (dyes **1–8**), with the selenophene-substituted reporters producing stronger SERS spectra than the thiophene-substituted dyes. The SERS spectra for dyes **1–13** were acquired with a 7 s acquisition time with the 1280 nm laser. The SERS spectrum of dye **14** with four 2-selenophenyl substituents was collected with only 3 s acquisition time due to the signal intensity saturating the spectrometer. Both **13** and **14** are significantly red-shifted with light absorption maxima >800 nm, making them NIR active. Dye **13** with four 2-thienyl substituents gave a weaker SERS signal compared to dye **14** with four 2-selenophenyl substituents. This suggests that the selenophene group adheres more effectively to the gold surface than thiophene and supports previous reports where selenolates have shown a greater affinity for gold surfaces than thiolates.^24^

The third component in the SERS nanotag is the aggregating agent, usually a simple inorganic salt such as KCl that screens the Coloumbic repulsion energy between the nanoparticles, allowing the reporter molecules to adhere more closely to the nanoparticle surface.^25^ Although the aggregating agent with an optimum concentration at 30 mM was necessary for most of the dyes, it is important to note that with dyes **13** and **14**, KCl was not required for intense signals to be observed (Fig. S4, ESI†). This is possibly due to a strong interaction occurring between the reporter and HGN surface inducing self-aggregation. This partial aggregation observed from these nanotags perhaps widens the scope for future SERS applications where aggregating agents are not required and the aggregation of the nanoparticles comes solely from a biological recognition event such as DNA–DNA interactions, DNA-protein interactions, peptide-protein interactions or sugar–protein interactions.^20,26^ These nanotags could be used as alternative reporters in biological applications such as photothermal ablation therapy or optical coherence tomography where there is a great need for NIR active materials.

### X-ray crystallographic studies of trimethine dye **14**

X-ray structural studies have shown that the chalcogenopyrylium/chalcogenopyranyl rings and the methine carbon of chalcogenopyrylium monomethine dyes related to **1–8** are coplanar and computational studies predict similar coplanarity in trimethine dyes **9–14**.^15*b*^ In the chalcogeno-pyrylium dyes bearing phenyl substituents at the 2-,2-′,6-, and 6′-positions, the phenyl rings for steric reasons cannot be coplanar with the chalcogenopyrylium/chalcogenopyranyl rings and the methine carbon. Other studies have shown that a 2-thienyl group can be coplanar with an attached thiopyranyl ring.^27^ By having reduced steric interactions with an attached pyranyl ring, the 2-thienyl substituent has more conformational freedom to optimize interactions with a gold surface to which it is attached in the monomethine and trimethine dyes of this study.

**Fig. 3:**
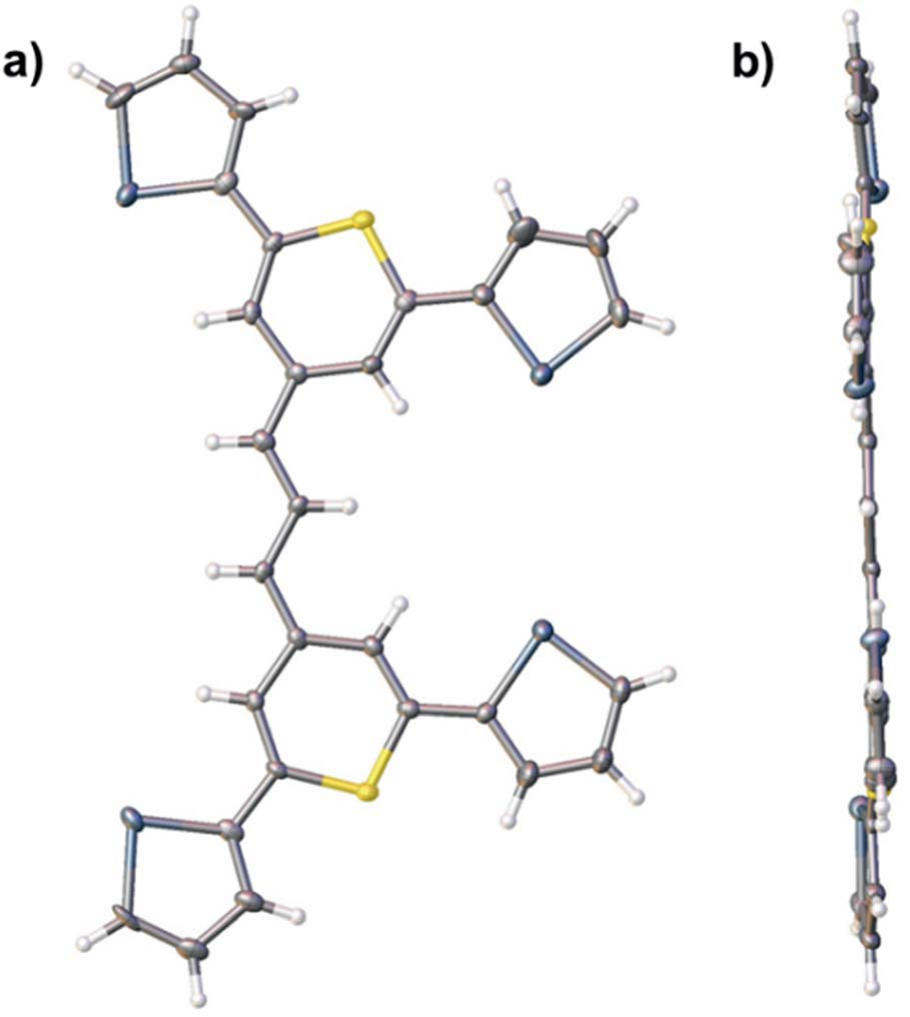
Thermal ellipsoid plots of the primary disorder species of dye **14** at the 50% probability level viewed (a) from above and (b) approximately parallel to the molecular plane. Atom colors are as follows: Se (dark grey), S (yellow), C (light grey), and H (white). Solvent molecules and counter-ion have been omitted for clarity.

X-ray crystallographic analysis of single crystals of dye **14** indicate that the thiopyrylium/thiopyranyl trimethine core and the four 2-selenophenyl substituents are highly coplanar as shown in Fig. 3 (angles between the mean-planes of the aromatic groups are given in the ESI†). In essence, all six chalcogen atoms can be involved in binding the reporter to the gold surface. Furthermore, the 2-selenophenyl substituents can rotate from coplanarity with the thiopyrylium/thiopyranyl trimethine core to a conformation to optimize binding to the HGN surface.

### Limits of detection (LODs) with reporter dyes **1–14**

Due to the exceptional response obtained with dye **14** and HGNs, particle dilution studies were conducted in order to calculate a limit of detection (LOD) for this dye at this extremely red-shifted laser wavelength. The LOD study was carried out by initially using the optimum conditions (those used in Fig. 1a to obtain SERS at 1280 nm and detailed in the ESI†) in which the dye concentration was 1.93 nM and then subsequent dilutions in water were made until no signal from dye **14** was observed. The peak at 1590 cm^–1^, which should arise from heterocyclic aromatic ring stretching within the molecule,^28^ was used to calculate the LOD since it was the most intense peak in the spectrum. Fig. 1b shows that a linear response was followed and a LOD of 1.5 ± 0.1 pM was calculated. The LOD was calculated to be 3 times the standard deviation of the blank, divided by the gradient of the straight line in Fig. 1b. LODs for dyes **1–13** were also measured and are compiled in Table 1. Trimethine dyes **9–13** all gave LODs of <10 pM with LODs of 1.8 ± 0.2 and 1.5 ± 0.1 pM, respectively, for **11** and **13**, which were comparable to dye **14**. Monomethine dyes **5**, **7**, and **8** gave LODs of 3.4 to 5.4 pM, respectively, while monomethine dyes **1–3** gave LODs of 22 to 33 pM. The 1280 nm SERS signals from reporters **4** and **6** were too weak to allow determination of LODs.

The non-resonant commercial dyes BPE (1,2-bis(4-pyridyl)ethylene) and AZPY (4,4-azopyridine), which are commonly used with gold nanosubstrates for SERS analysis^17b,29^ were also tested with the HGNs at this laser wavelength but failed to produce a SERS signal (Fig. S5, ESI†).

### Principal component analysis (PCA) with reporter dyes **1–14**

Dyes **1–14** can be separated out and individually identified in a reproducible manner based on their unique structures and SERS spectra through multivariate analysis in the form of principal component analysis (PCA). PCA is employed to reduce the dimensionality of the spectroscopic data thus facilitating identification of variations in the SERS spectra.^30^

**Fig. 4:**
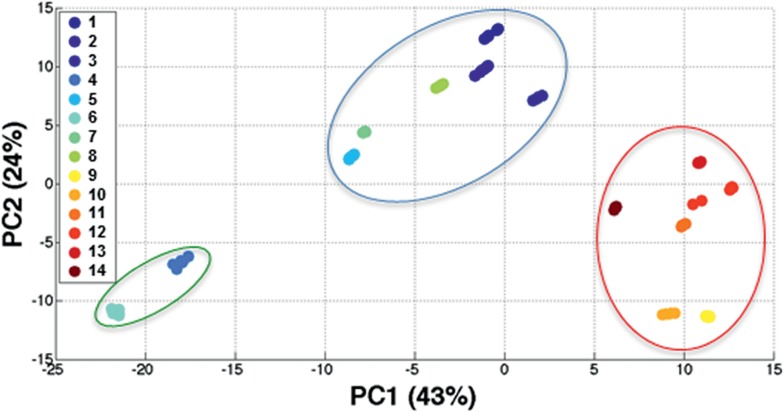
PCA scores plot discriminating between each of the 14 chalcogenopyrylium dyes and grouping them according to their structures and SERS spectra. The red cluster contains the trimethine dyes **9–14** which produce the best SERS signals, blue cluster highlights the monomethine dyes (**1–3**,**5**,**7**,**8**) which work well as reporters for SERS and the green clustering contains the two dyes which produce only weak signals with HGNs (dyes **4** and **6**).

PCA was carried out on 14 data sets consisting of three replicate spectra obtained from each individual dye. The resulting principal component (PC) scores plot (Fig. 4) clearly illustrates three unique groupings. The red cluster contains the trimethine dyes **9–14**; these reporter molecules produced the most intense SERS signals and all contain 3 sp^2^ carbons in their trimethine bridge. The blue clustering highlights the monomethine dyes (**1–3**,**5**,**7**,**8**), which are good Raman reporters and produce intense SERS spectra with HGNs and KCl while the green cluster contains the two dyes which produced a weak SERS response (dyes **4** and **6**) when excited with the 1280 nm laser. The monomethine dyes only contain 1 sp^2^ carbon in their monomethine bridge and this simple difference in molecular structure could be responsible for the variation in signal intensities observed between the trimethine and monomethine dyes. Moreover, this simple structural change can affect the distance, orientation and/or the polarizability of the reporter which ultimately affects the SERS response. Loadings plots showing the spectral variations responsible for the classification of the dyes can be viewed in Fig. S6, ESI.†

Additionally, it can be observed that within the three groupings, all 14 dyes can be individually identified by PCA and classified according to their unique structure and SERS spectra. Replicates for each dye are tightly clustered illustrating the excellent reproducibility of the SERS spectra. A further benefit for these 1280 nm SERS nanotags is their potential for use in future multiplexing systems, where multiple analytes need to be identified simultaneously, such as in chemical or medical detection assays.^26d,31^

## Conclusions

In conclusion, a set of new extreme red shifted SERS nanotags have been designed and synthesized to demonstrate unprecedented performance using 1280 nm excitation, a set of chalcogenopyrylium dye reporter molecules, and HGNs. These nanotags show a LOD in the picomolar range. The dye molecules are unique NIR reporters as they have multiple chalcogen attachment groups which allow them to bind strongly to the gold surface of the HGN and thus produce intense SERS signals. Dyes **1–14** with the more widely used gold nanoparticles or HGNs with conventional Raman reporters such as BPE were unable to match the combined performance of the chalcogenopyrylium dyes and HGNs indicating the unexpected and superior performance of SERS nanotags based on the combination of these dyes and the tunable HGNs. This significant result now makes SERS nanotags available for future use in a wide range of optical applications with 1280 nm excitation including deep tissue analysis and will provide a basis for future studies into harnessing their unique spectral properties.
