# Supplementary material for: Extreme red shifted SERS nanotags
Source: Chem Sci. 2015 Jan 21;6(4):2302–6. doi: 10.1039/c4sc03917c (PMC5645778; doi:10.1039/c4sc03917c)
Supplement: Supplementary file 1 [file SC-006-C4SC03917C-s001.pdf]

Electronic Supplementary Material (ESI) for Chemical Science.
This journal is © The Royal Society of Chemistry 2015

Supporting Information

Extreme Red Shifted SERS Nanotags

Matthew A. Bedics,[†] Hayleigh Kearns,[‡] Jordan Cox,[†] Sam Mabbott,[‡] Fatima Ali,[‡] Neil C. Shand,[§] Karen Faulds,[‡] Jason B. Benedict,[†] Duncan Graham,[‡,*] and Michael R. Detty[†,*]

† Department of Chemistry, University at Buffalo, The State University of New York, New York 14260, United States

‡ Centre for Molecular Nanometrology, WestCHEM, Department of Pure and Applied Chemistry, University of Strathclyde, 295 Cathedral Street, Glasgow, G1 1XL, United Kingdom

§ Dstl, Porton Down, Salisbury, UK

Table of Contents

General Experimental S3

Scheme S1. Synthesis of thiopyranone **16a** and 4-methylpyrylium salt **15c** S3

Detailed Experimental S3-S14

Scheme S2. Synthesis of chalcogenopyrylium dyes **1-14** S5

Synthesis of HGNs S14

Characterisation of HGNs S14

Chemometrics S15

Figure S1. SERS spectra and structures of dyes **1-14** S16-S20

Figure S2. a) Extinction spectrum showing the SPR of the HGNs at 720 nm

b) Extended extinction spectra showing that the SPR does not shift upon aggregation. S20

Figure S3. SERS spectra for dye **13** with gold and silver nanoparticles S21

Figure S4. Unaggregated SERS spectra of dyes **13** and **14** analyzed with HGNs S21

Figure S5. SERS spectra and structures of BPE and AZPY with HGNs S22

Figure S6. PC loading plots S22

Figure S7. SERS particle dilution studies for dyes **1-13** S23-S24

References S25

Crystallographic details of dye **14** S26

Figure S8. Calculated angles between mean planes in dye **14** S26

Table S1. Crystal data and structure refinement for dye **14** S27

Table S2. Fractional Atomic Coordinates ($\times10^4$) and Equivalent Isotropic Displacement Parameters (Å$^2\times10^3$) for **14** S28

Table S3. Anisotropic Displacement Parameters (Å$^2\times10^3$) for **14** S30

Table S4. Bond Lengths for **14** S32

Table S5. Bond Angles for **14** S33

Table S6. Torsion angles for **14** S35

Table S7. Atomic occupancy for dye **14** S37

Crystallographic references S37

General Experimental

All reactions were performed open to air unless otherwise noted. Concentration *in vacuo* was performed on a rotary evaporator. NMR spectra were recorded at 300 or 500 MHz for ^{1}H and at 75.5 MHz for ^{13}C with residual solvent signal as internal standard. UV/VIS-near-IR spectra were recorded in quartz cuvettes with a 1-cm path length. Melting points were determined with a capillary melting point apparatus and are uncorrected. Non-hygroscopic compounds have a purity of ≥ 95% as determined by elemental analyses for C, H, and N. Experimental values of C, H, and N are within 0.3% of theoretical values. ^{13}C NMR spectra were not recorded for pyrylium dyes due to limited solubility in common NMR solvents. Pyranones **16b-16d** are known compounds.[S1] The synthesis of thiopyranone **16a** and 4-methylpyrylium salt **15c** is shown in Scheme S1. 4-Methylpyrylium salts **15a**, **15d**, and **15e** have been reported previously in the literature.[S2-S4]

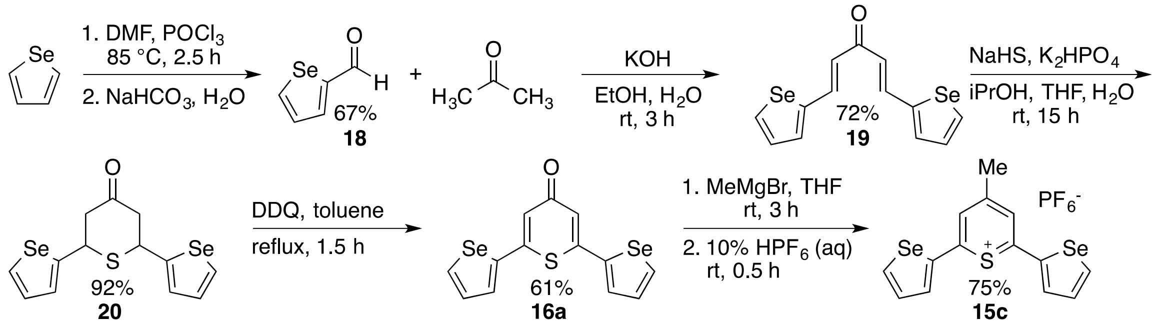

Scheme S1. Synthesis of thiopyranone **16a** and 4-methylpyrylium salt **15c**.

Detailed Experimental

Synthesis of selenophen-2-carboxaldehyde (18)[S5]

Anhydrous DMF (15.0 mL) was added to a flame dried flask under argon and cooled to 0 °C. $POCl_3$ (1.71 mL, 18.3 mmol) was added and the mixture allowed to stir for 0.5 h. The ice bath was removed, and selenophene (1.41 mL, 15.3 mmol) added. The reaction was heated to 85 °C and maintained at this temperature for 2.5 h. After cooling to ambient temperature, the mixture was poured into ice water (200 mL), neutralized with satd. $NaHCO_3$ (100 mL) and the product extracted with EtOAc (2 × 75 mL). The organic layer was dried with $MgSO_4$ and after concentration purified on SiO_2 with a 25% EtOAc/hexanes eluent to yield 1.64 g (67%) of a

colorless oil: ^{1}H NMR [500 MHz, $CDCl_3$] δ 9.83 (s, 1 H), 8.50 (d, 1 H, *J* = 5.5 Hz), 8.03 (d, 1 H, *J* = 4.0 Hz), 7.49-7.47 (m, 1 H).

Synthesis of (1*E*,4*E*)-1,5-di(selenophen-2-yl)penta-1,4-dien-3-one (19)

Selenophen-2-carbaldehyde (1.64 g, 10.3 mmol) and acetone (0.376 mL, 5.13 mmol) were dissolved in EtOH (10 mL). KOH (0.287 g, 5.13 mmol) dissolved in H_2O was added slowly to the stirring mixture and then allowed to stir for 3 h at ambient temperature. The solution was diluted with H_2O (50 mL), and the product extracted with CH_2Cl_2 (2 × 50 mL). The organic layer was dried with $MgSO_4$, concentrated, and recrystallized from CH_2Cl_2/hexanes to yield 1.26 g (72%) of a yellow crystalline solid, mp 135-137 °C: ^{1}H NMR [500 MHz, $CDCl_3$] δ 8.09 (d, 2 H, *J* = 5.5 Hz), 7.85 (d, 2 H, *J* = 15.5 Hz), 7.52 (d, 2 H, *J* = 3.5 Hz), 7.30 (m, 2 H), 6.70 (d, 2 H, *J* = 15.0 Hz); ^{13}C NMR [75.5 MHz, $CDCl_3$] δ 187.5, 146.2, 137.8, 135.1, 133.9, 130.7, 125.7; HRMS (ESI, HRDFMagSec) *m/z* 342.9131 (calcd for $C_{13}H_{10}O^{80}Se_2+H^+$: 342.9135).

Synthesis of 2,6-di(selenophen-2-yl)tetrahydro-4*H*-thiopyran-4-one (20)

(1*E*,4*E*)-1,5-Di(selenophen-2-yl)penta-1,4-dien-3-one (1.20 g, 3.51 mmol) was dissolved in THF (5.0 mL). To this mixture isopropyl alcohol (10 mL), and K_2HPO_4 (0.961 g, 4.21 mmol) dissolved in H_2O were added followed by the addition of NaHS (0.356 g, 3.86 mmol). This was allowed to stir overnight at ambient temperature and under an argon atmosphere. The reaction was then diluted with H_2O (50 mL) and the product extracted with CH_2Cl_2 (2 × 50 mL). The organic layer was dried with $MgSO_4$, concentrated and then recrystallized from CH_2Cl_2/hexanes to yield 1.21 g (92%) of an off-white solid, mp 115-116 °C: ^{1}H NMR [300 MHz, $CDCl_3$] δ 7.97 (dd, 2 H, *J* = 5.3, 2.0 Hz), 7.21-7.17 (m, 4 H), 4.71 (dd, 2 H, *J* = 12.3, 5.0 Hz), 3.14 (dd, 2 H, *J* = 13.8, 2.7 Hz), 2.95 (m, 2 H); ^{13}C NMR [75.5 MHz, $CDCl_3$] δ 205.6, 149.3, 130.9, 129.1, 127.1, 52.0, 45.5; HRMS (EI, HRDFMagSec) *m/z* 375.8933 (calcd for $C_{13}H_{12}OS^{80}Se_2$: 375.8934).

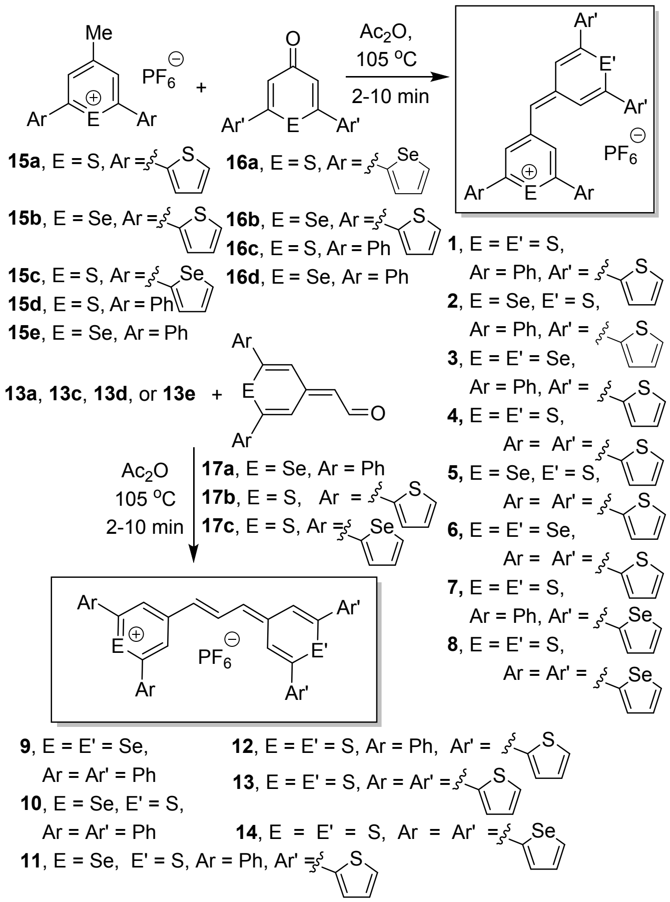

Scheme S2. Synthesis of chalcogenopyrylium dyes **1-14**.

Synthesis of 2,6-di(selenophen-2-yl)-4*H*-thiopyran-4-one (16a)

2,6-Di(selenophen-2-yl)tetrahydro-4*H*-thiopyran-4-one (0.450 g, 1.20 mmol) was dissolved in anhydrous toluene (6.0 mL) and placed in a flame-dried flask under argon. 2,3-Dichloro-5,6-dicyano-1,4-benzoquinone (0.679 g, 2.99 mmol) was added in one portion and the reaction refluxed for 1.5 h. This was cooled to ambient temperature, diluted with CH_2Cl_2 (50 mL) and the mixture washed with satd. aqueous $NaHCO_3$ (50 mL). The organic layer was separated, dried with $MgSO_4$, and after concentration purified on SiO_2 with a 20% EtOAc/CH_2Cl_2 eluent (R_f 0.47) to yield 0.273 g (61%) of a light brown solid, mp 138-140 °C: ^{1}H NMR [500 MHz, $CDCl_3$] δ 8.19 (dd, 2 H, *J* = 5.5, 1.5 Hz), 7.69 (dd, 2 H, *J* = 4.0, 1.5 Hz), 7.38 (m, 2 H), 7.09 (s, 2 H); ^{13}C NMR [75.5 MHz, $CDCl_3$] δ 182.1, 146.8, 143.6, 134.8, 130.8, 129.5, 125.7; HRMS (ESI, HRDFMagSec) *m/z* 372.8699 (calcd for $C_{13}H_8OS^{80}Se_2+H^+$: 372.8698).

Synthesis of 4-methyl-2,6-di(2-thienyl)thiopyrylium hexafluorophosphate (15a)

2,6-Bis(thiophen-2-yl)-4H-thiopyran-4-one (0.288 g, 1.04 mmol) was dissolved in anhydrous THF (7.0 mL) in a flame dried flask under argon. 3.0 M MeMgBr (1.04 mL, 3.12 mmol) was added dropwise to this solution and allowed to stir at ambient temperature for 2 h. The solution was poured into 10% aqueous HPF_6 (50 mL) and allowed to stir for 15 min before the solid was isolated by filtration. The resulting solid was dissolved in CH_2Cl_2, dried with Na_2SO_4, and the solvent removed under reduced pressure. The product was then recrystallized from CH_3CN/ether to yield 0.381 g (87%) of a bright red solid, mp 190-192 °C: ^1H NMR [500 MHz, CD_3CN] δ 8.35 (s, 2 H), 8.08-8.07 (m, 4 H), 7.39 (t, 2 H, *J* = 5.0 Hz), 2.78 (s, 3 H); ^{13}C NMR [75.5 MHz, CD_3CN] δ 167.6, 160.0, 137.9, 137.2, 134.0, 131.8, 131.2, 25.9; HRMS (ESI, HRDFMagSec) *m/z* 275.0021 (calcd for $C_{14}H_{11}S_3^+$: 275.0017).

Synthesis of 4-methyl-2,6-bis(2-thienyl)selenopyrylium hexafluorophosphate (15b)

In a flame-dried flask under argon, 2,6-bis(thiophen-2-yl)-4H-selenopyran-4-one (0.200 g, 0.621 mmol), was dissolved in anhydrous THF (5.0 mL). 3.0 M MeMgBr (0.620 mL, 1.86 mmol) was added dropwise and the solution allowed to stir at ambient temperature for 0.5 h. The mixture was quenched with MeOH (1 mL), poured into 10% aqueous HPF_6 (50 mL), and extracted with CH_2Cl_2 (3 × 25 mL). The organic layer was dried with Na_2SO_4, concentrated under reduced pressure, and then recrystallized from CH_3CN/ether to yield 0.239 g (82%) of a red solid, mp 185-187 °C: ^1H NMR [500 MHz, CD_3CN] δ 8.23 (s, 4 H), 8.11 (d, 2 H, *J* = 5.5 Hz), 8.02 (d, 2 H, *J* = 4.5 Hz), 7.39 (t, 2 H, *J* = 4.0 Hz), 2.70 (s, 3 H); ^{13}C NMR [75.5 MHz, CD_3CN] δ 168.2, 167.7, 139.7, 134.0, 132.10, 131.4, 27.3; HRMS (ESI, HRDFMagSec) *m/z* 322.9469 (calcd for $C_{14}H_{11}S_2{}^{80}Se^+$: 322.9462).

Synthesis of 4-methyl-2,6-di(selenophen-2-yl)thiopyrylium hexafluorophosphate (15c)

2,6-di(selenophen-2-yl)-4*H*-thiopyran-4-one (0.300 g, 0.807 mmol) was dissolved in anhydrous THF (8.0 mL) in a flame-dried flask under argon. 3.0 M MeMgBr (0.807 mL, 2.42 mmol) was added dropwise and the reaction stirred at ambient temperature for 1 h. The reaction was poured into 10% aqueous HPF_6 (40 mL), and stirred for 10 min. The resulting solid was extracted with a mixture of CH_2Cl_2 (50 mL) and CH_3CN (5.0 mL), dried with Na_2SO_4, and after concentration recrystallized from CH_3CN/ether to yield 0.312 g (75%) of a bright red solid, mp >260 °C: ^1H NMR [500 MHz, CD_3CN] δ 8.78 (d, 2 H, *J* = 5.5 Hz), 8.26-8.25 (m, 4 H), 7.61 (t, 2 H, *J* = 4.5

Hz), 2.76 (s, 3 H); ^{13}C NMR [75.5 MHz, $CDCl_3$] δ 167.4, 162.2, 145.0, 142.1, 136.6, 133.9, 132.2, 25.8; HRMS (EI, HRDFMagSec) *m/z* 370.8900 (calcd for $C_{14}H_{11}S^{80}Se_2^+$: 370.8906).

Synthesis of 4-(2,6-diphenyl-4H-thiopyran-4ylidene)acetaldehyde (17a)

4-Methyl-2,6-di(phenyl)selenopyrylium hexafluorophosphate (0.200 g, 0.439 mmol), *N,N*-dimethylthioformamide (0.112 mL, 1.32 mmol) and Ac_2O (4.0 mL) were added to a round-bottom flask and heated at 95 °C for 90 min. After cooling to ambient temperature CH_3CN (4.0 mL) was added and the product precipitated by addition of ether and chilling overnight in the freezer. The iminium salt was isolated by filtration, and hydrolyzed by dissolving in CH_3CN (4.0 mL), adding satd. aqueous $NaHCO_3$ (4.0 mL) and heating the mixture to 80 °C over a 15 min period. The reaction was maintained at this temperature for 0.5 h, after which the reaction was diluted with H_2O (50 mL), the product extracted with CH_2Cl_2 (3 × 30 mL), dried with $MgSO_4$, and after concentration purified on SiO_2 with first a CH_2Cl_2 and then a 10% EtOAc/CH_2Cl_2 (R_f = 0.70) eluent to yield 0.122 g (82%) of a orange oil: ^1H NMR [500 MHz, $CDCl_3$] δ 10.11 (d, 1 H, *J* = 10.5 Hz), 8.32 (s, 1 H), 7.62-7.46 (m, 9 H), 7.00 (s, 1 H), 5.88 (d, 1 H, *J* = 11.0 Hz); ^{13}C NMR [75.5 MHz, $CDCl_3$] δ 188.7, 148.4, 147.0, 146.0, 138.7, 138.4, 130.0, 129.9, 129.1, 126.6, 126.4, 126.4, 126.3, 125.7, 120.6, 120.5; HRMS (EI, HRDFMagSec) *m/z* 339.0292 (calcd for $C_{19}H_{14}O^{80}Se + H^+$: 339.0283).

Synthesis of 4-(2,6-di(thiophene-2-yl)-4H-thiopyran-4ylidene)acetaldehyde (17b)

4-Methyl-2,6-di(thiophen-2-yl)thiopyrylium hexafluorophosphate (0.350 g, 0.833 mmol), *N,N*-dimethylthioformamide (0.213 mL, 2.50 mmol) and Ac_2O (3.0 mL) were combined in a small round bottom flask and heated at 95 °C for 1 h. After cooling to ambient temperature an additional portion of Ac_2O (2.0 mL) was added and the solution diluted with ether. The formed iminium salt was allowed to precipitate in the freezer overnight, and then isolated by filtration to yield a bright orange solid. This solid was dissolved in CH_3CN (3.0 mL) and satd. aqueous $NaHCO_3$ (3.0 mL) was added. This mixture was heated to 80 °C over 15 min, and kept at that temperature for 0.5 h. After diluting with H_2O (30 mL) the product was extracted with CH_2Cl_2 (3 × 50 mL), dried with Na_2SO_4 and purified on SiO_2 with a 10% EtOAc/CH_2Cl_2 eluent (R_f = 0.71) to yield a yellow oil that was recrystallized in CH_2Cl_2/hexanes to yield 0.219 g (87%) of a yellow crystalline solid, mp 143-144 °C: ^1H NMR [500 MHz, $CDCl_3$] δ 9.84 (d, 1 H, *J* = 6.0 Hz), 8.26 (s, 1 H), 7.45-7.39 (m, 4 H), 7.13-7.11 (m, 2 H), 6.88 (s, 1 H), 5.72 (d, 1 H, *J* = 6.5 Hz); ^{13}C

NMR [75.5 MHz, $CDCl_3$] δ 188.1, 146.4, 139.4, 139.1, 137.3, 136.7, 128.2, 127.8, 127.6, 126.3, 126.0, 122.5, 117.6, 117.5; HRMS (EI, HRDFMagSec) *m/z* 302.9971 (calcd for $C_{15}H_{10}OS_3$+ H^+: 302.9967).

Synthesis of 2-(2,6-di(selenophen-2-yl)-4*H*-thiopyran-4-ylidene)acetaldehyde (17c)

4-Methyl-2,6-di(selenophen-2-yl)thiopyrylium hexafluorophosphate (0.150 g, 0.291 mmol), *N,N*-dimethylthioformamide (74.3 µL, 0.872 mmol) and Ac_2O (2.0 mL) were added to a round-bottom flask and heated at 95 °C for 90 min. After cooling to ambient temperature, Ac_2O (2.0 mL) was added and the product precipitated by addition of ether and chilling overnight in the freezer. The iminium salt was isolated by filtration, and hydrolyzed by dissolving in CH_3CN (3.0 mL), adding satd. aqueous $NaHCO_3$ (3.0 mL) and heating the mixture to 80 °C over a 15 min period. The reaction was maintained at this temperature for ½ h, after which the reaction was diluted with H_2O (30 mL), the product extracted with CH_2Cl_2 (3 × 20 mL), dried with $MgSO_4$, and after concentration purified on SiO_2 with a 10% EtOAc/CH_2Cl_2 (R_f = 0.62) eluent to yield 80.3 mg (69%) of a brown solid, mp 145-146 °C: ^1H NMR [500 MHz, $CDCl_3$] δ 9.95 (d, 1 H, *J* = 6.0 Hz), 8.21 (s, 1 H), 8.13-8.10 (m, 2 H), 7.62 (d, 1 H, *J* = 4.0 Hz), 7.57 (d, 1 H, *J* = 4.0 Hz), 7.36-7.33 (m, 2 H), 6.81 (s, 1 H), 5.72 (d, 1 H, *J* = 6.0 Hz); ^{13}C NMR [75.5 MHz, $CDCl_3$] δ 188.1, 146.7, 144.9, 144.6, 139.5, 138.8, 133.5, 133.2, 130.6, 128.6, 128.3, 123.3, 118.5, 117.4; HRMS (ESI, HRDFMagSec) *m/z* 398.8861 (calcd for $C_{15}H_{10}OS^{80}Se_2$+H^+ : 398.8856).

Synthesis of 4-((2,6-diphenyl-4*H*-thiopyran-4-ylidene)methyl)-2,6-di(thiophen-2-yl)thiopyrylium hexafluorophosphate (1)

4-Methyl-2,6-di(thiophen-2-yl)thiopyrylium hexafluorophosphate (50.0 mg, 0.119 mmol), 2,6-bis(phenyl)-4H-thiopyran-4-one (34.6 mg, 0.131 mmol) and Ac_2O (2.0 mL) were heated for 5 min at 105 °C prior to cooling to ambient temperature, diluting with CH_3CN (3.0 mL) and adding ether to precipitate the product. Yielded 77.6 mg (91%) of a copper bronze solid, mp 222-223 °C: ^1H NMR [500 MHz, CD_2Cl_2] δ 8.04 (br s, 2 H), 7.90 (br s, 2 H), 7.83 (d, 4 H, *J* = 8.0 Hz), 7.75-7.73 (m, 4 H), 7.70-7.63 (m, 6 H), 7.30 (t, 2 H, *J* = 4.5 Hz), 6.67 (s, 1 H); λ_{max} (CH_2Cl_2) = 653 nm (ε 1.3 x 10^5 $M^{-1}cm^{-1}$); 473 nm (ε 1.6 × 10^4 $M^{-1}cm^{-1}$); 410 nm (ε 1.4 × 10^4 $M^{-1}cm^{-1}$); HRMS (ESI, HRDFMagSec) *m/z* 521.0518 (calcd for $C_{31}H_{21}S_4^+$: 521.0521). **Anal.** Calcd for $C_{31}H_{21}S_4 \cdot PF_6$: C, 55.85; H, 3.17. Found: C, 55.90; H, 3.29.

Synthesis of 4-((2,6-diphenyl-4*H*-selenopyran-4-ylidene)methyl)-2,6-di(thiophen-2-yl)thiopyrylium hexafluorophosphate (2)

4-Methyl-2,6-di(thiophen-2-yl)thiopyrylium hexafluorophosphate (50.0 mg, 0.119 mmol), 2,6-bis(phenyl)-4H-selenopyran-4-one (40.6 mg, 0.131 mmol) and Ac_2O (2.0 mL) were heated for 5 min at 105 °C prior to cooling to ambient temperature, diluting with CH_3CN (3.0 mL) and adding ether to precipitate the product. Yielded 77.6 mg (91%) of a copper bronze solid, mp 249-250 °C: ^1H NMR [500 MHz, CD_2Cl_2] δ 8.02 (br s, 2 H), 7.94 (s, 2 H), 7.78-7.75 (m, 8 H), 7.67-7.60 (m, 6 H), 7.30 (t, 2 H, J = 5.0 Hz), 6.76 (s, 1 H); λ_{max} (CH_2Cl_2) = 676 nm (ε 1.3×10^5 $M^{-1}cm^{-1}$); 483 nm (ε 1.6×10^4 $M^{-1}cm^{-1}$); 422 nm (ε 1.5×10^4 $M^{-1}cm^{-1}$); HRMS (ESI, HRDFMagSec) *m/z* 568.9958 (calcd for $C_{31}H_{21}S_3{}^{80}Se^+$: 568.9965). **Anal.** Calcd for $C_{31}H_{21}S_3Se{\cdot}PF_6{\cdot}\frac{1}{2}H_2O$: C, 51.53; H, 3.07. Found: C, 51.55; H, 3.01.

Synthesis of 4-((2,6-diphenyl-4*H*-selenopyran-4-ylidene)methyl)-2,6-di(thiophen-2-yl)selenopyrylium hexafluorophosphate (3)

4-Methyl-2,6-di(thiophen-2-yl)selenopyrylium hexafluorophosphate (50.0 mg, 0.107 mmol), 2,6-bis(phenyl)-4H-selenopyran-4-one (36.6 mg, 0.118 mmol) and Ac_2O (4.0 mL) were heated for 90 sec at 105 °C prior to cooling to ambient temperature, diluting with CH_3CN (3.0 mL) and adding ether to precipitate the product. Product was purified on SiO_2 with a 10% EtOAc/CH_2Cl_2 eluent (R_f = 0.34) to yield 35.7 mg (44%) of a copper bronze solid, mp 243-244 °C: ^1H NMR [500 MHz, CD_2Cl_2] δ 8.06 (br s, 2 H), 7.93 (s, 2 H), 7.79 (d, 4 H, J = 7.5 Hz), 7.75 (d, 2 H, J = 5.5 Hz), 7.68-7.65 (m, 4 H), 7.62 (t, 4 H, J = 7.0 Hz), 7.29 (t, 2 H, J = 4.5 Hz), 6.86 (s, 1 H); λ_{max} (CH_2Cl_2) = 699 nm (ε 1.5×10^5 $M^{-1}cm^{-1}$); 499 nm (ε 1.9×10^4 $M^{-1}cm^{-1}$); 428 nm (ε 1.8×10^4 $M^{-1}cm^{-1}$); HRMS (ESI, HRDFMagSec) *m/z* 616.9402 (calcd for $C_{31}H_{21}S_2{}^{80}Se_2{}^+$: 616.9410). **Anal.** Calcd for $C_{31}H_{21}S_2Se_2{\cdot}PF_6$: C, 48.96; H, 2.78. Found: C, 48.68; H, 2.76.

Synthesis of 4-((2,6-di(thiophen-2-yl)-4*H*-thiopyran-4-ylidene)methyl)-2,6-di(thiophen-2-yl)thiopyrylium hexafluorophosphate (4)

4-Methyl-2,6-di(thiophen-2-yl)thiopyrylium hexafluorophosphate (50.0 mg, 0.119 mmol), 2,6-bis(thiophen-2-yl)-4H-thiopyran-4-one (36.2 mg, 0.131 mmol) and Ac_2O (2.0 mL) were heated for 20 min at 105 °C prior to cooling to ambient temperature, diluting with CH_3CN (3.0 mL) and

adding ether to precipitate the product. Yielded 78.6 mg (97%) of a copper bronze solid, mp >260 °C: ^{1}H NMR [500 MHz, CD_2Cl_2] δ 7.86 (br s, 4 H), 7.75 (d, 8 H, *J* = 4.5 Hz), 7.30 (t, 4 H, *J* = 4.5 Hz), 6.61 (s, 1 H); λ_{max} (CH_2Cl_2) = 676 nm (ε 1.2 × 10^5 $M^{-1}cm^{-1}$); 480 nm (ε 2.7 × 10^4 $M^{-1}cm^{-1}$); HRMS (ESI, HRDFMagSec) *m/z* 532.9628 (calcd for $C_{27}H_{17}S_6^+$: 532.9649). **Anal.** Calcd for $C_{27}H_{17}S_6{\cdot}PF_6$: C, 47.78; H, 2.52. Found: C, 47.94; H, 2.44.

Synthesis of 4-((2,6-di(thiophen-2-yl)-4*H*-thiopyran-4-ylidene)methyl)-2,6-di(thiophen-2-yl)selenopyrylium hexafluorophosphate (5)

4-Methyl-2,6-di(thiophen-2-yl)thiopyrylium hexafluorophosphate (50.0 mg, 0.119 mmol), 2,6-bis(thiophen-2-yl)-4H-selenopyran-4-one (42.3 mg, 0.131 mmol) and Ac_2O (2.0 mL) were heated for 5 min at 105 °C prior to cooling to ambient temperature, diluting with CH_3CN (3.0 mL) and adding ether to precipitate the product. Yielded 83.2 mg (96%) of a copper bronze solid, mp 254-256 °C: ^{1}H NMR [500 MHz, CD_2Cl_2] δ 7.94 (s, 2 H), 7.89 (br s, 2 H), 7.83-7.87 (m, 6 H), 7.71 (dd, 2 H, *J* = 4.0, 1.0 Hz), 6.73 (s, 1 H); λ_{max} (CH_2Cl_2) = 698 nm (ε 1.1 × 10^5 $M^{-1}cm^{-1}$); 493 nm (ε 2.5 × 10^4 $M^{-1}cm^{-1}$); HRMS (ESI, HRDFMagSec) *m/z* 580.9087 (calcd for $C_{27}H_{17}S_5{}^{80}Se^+$: 580.9094). **Anal.** Calcd for $C_{27}H_{17}S_5Se{\cdot}PF_6$: C, 44.69; H, 2.36. Found: C, 44.76; H, 2.49.

Synthesis of 4-((2,6-di(thiophen-2-yl)-4*H*-selenopyran-4-ylidene)methyl)-2,6-di(thiophen-2-yl)selenopyrylium hexafluorophosphate (6)

4-Methyl-2,6-di(thiophen-2-yl)selenopyrylium hexafluorophosphate (50.0 mg, 0.107 mmol), 2,6-bis(thiophen-2-yl)-4H-selenopyran-4-one (37.9 mg, 0.118 mmol) and Ac_2O (2.0 mL) were heated for 5 min at 105 °C prior to cooling to ambient temperature, diluting with CH_3CN (3.0 mL) and adding ether to precipitate the product. Yielded 77.6 mg (94%) of a copper bronze solid, mp 233-234 °C: ^{1}H NMR [500 MHz, CD_2Cl_2] δ 7.92 (br s, 4 H), 7.79 (d, 4 H, *J* = 5.0 Hz), 7.74 (d, 4 H, *J* = 3.0 Hz), 7.33 (t, 4 H, *J* = 4.5 Hz), 6.82 (s, 1 H); λ_{max} (CH_2Cl_2) = 723 nm (ε 1.3 × 10^5 $M^{-1}cm^{-1}$); 506 nm (ε 2.9 × 10^4 $M^{-1}cm^{-1}$); HRMS (ESI, HRDFMagSec) *m/z* 628.8533 (calcd for $C_{27}H_{17}S_4{}^{80}Se_2^+$: 628.8543). **Anal.** Calcd for $C_{27}H_{17}S_4Se_2{\cdot}PF_6$: C, 41.98; H, 2.22. Found: C, 41.69; H, 2.15.

Synthesis of 4-((2,6-diphenyl-4*H*-thiopyran-4-ylidene)methyl)-2,6-di(selenophen-2-yl)thiopyrylium hexafluorophosphate (7)

4-Methyl-2,6-diphenylthiopyrylium hexafluorophosphate (50.0 mg, 0.123 mmol), 2,6-di(selenophen-2-yl)-4*H*-thiopyran-4-one (50.1 mg, 0.135 mmol) and Ac_2O (2.0 mL) were heated for 5 min at 105 °C prior to cooling to ambient temperature, diluting with CH_3CN (3.0 mL) and adding ether to precipitate the product to yield 79.0 mg (85%) of a copper bronze solid, mp > 260 °C: ^{1}H NMR [500 MHz, CD_2Cl_2] δ 8.44 (d, 2 H, J = 5.0 Hz), 8.03 (br s, 2 H), 7.91 (d, 2 H, J = 4.0 Hz), 7.83-7.82 (m, 6 H), 7.69-7.62 (m, 6 H), 7.51 (t, 2 H, J = 5.0 Hz), 6.72 (s, 1 H); λ_{max} (CH_2Cl_2) = 659 nm (ε 1.4 × 10^5 $M^{-1}cm^{-1}$); 484 nm (ε 1.8 × 10^4 $M^{-1}cm^{-1}$); 428 nm (ε 1.7 × 10^4 $M^{-1}cm^{-1}$); HRMS (ESI, HRDFMagSec) *m/z* 616.9397 (calcd for $C_{31}H_{21}S_2{}^{80}Se_2{}^+$: 616.9410). **Anal.** Calcd for $C_{31}H_{21}S_2Se_2{\cdot}PF_6$: C, 48.96; H, 2.78. Found: C, 49.09; H, 2.98.

Synthesis of 4-((2,6-di(selenophen-2-yl)-4*H*-thiopyran-4-ylidene)methyl)-2,6-di(selenophen-2-yl)thiopyrylium hexafluorophosphate (8)

4-Methyl-2,6-di(selenophen-2-yl)thiopyrylium hexafluorophosphate (50.0 mg, 96.9 μmol), 2,6-di(selenophen-2-yl)-4*H*-thiopyran-4-one (39.6 mg, 0.107 mmol) and Ac_2O (2.0 mL) were heated for 5 min at 105 °C prior to cooling to ambient temperature, diluting with CH_3CN (3.0 mL) and adding ether to precipitate the product to yield 64.2 mg (76%) of a copper bronze solid, mp > 260 °C: ^{1}H NMR [500 MHz, CD_3CN] δ 8.48 (d, 4 H, J = 6.0 Hz), 7.93 (d, 4 H, J = 3.5 Hz), 7.74 (s, 4 H), 7.47 (t, 4 H, J = 5.0 Hz), 6.57 (s, 1 H); λ_{max} (CH_2Cl_2) = 687 nm (ε 1.1 × 10^5 $M^{-1}cm^{-1}$); 491 nm (ε 2.8 × 10^4 $M^{-1}cm^{-1}$); HRMS (ESI, HRDFMagSec) *m/z* 724.7393 (calcd for $C_{27}H_{17}S_2{}^{80}Se_4{}^+$: 724.7427). **Anal.** Calcd for $C_{27}H_{17}S_2Se_4{\cdot}PF_6$: C, 37.43; H, 1.98. Found: C, 37.70; H, 2.06.

Synthesis of 4-(3-(2,6-diphenyl-4H-selenopyran-4-ylidene)prop-1-enyl)-2,6-diphenylselenopyrylium hexafluorophosphate (9)[S4]

4-Methyl-2,6-di(phenyl)selenopyrylium hexafluorophosphate (0.190 g, 0.417 mmol), 4-(2,6-diphenyl-4H-selenopyran-4ylidene)acetaldehyde (0.155 g, 0.459 mmol) and Ac_2O (3.0 mL) were combined in a round bottom flask and heated at 105 °C for 10 min. The reaction was cooled to ambient temperature, precipitated with ether, and the collected solid recrystallized from

CH_3CN/ether to yield 0.278 g (86%) of a golden-green solid: ^{1}H NMR [500 MHz, CD_2Cl_2] δ 8.59 (t, 1 H, *J* = 13.5 Hz), 8.40-7.80 (br s, 4 H), 7.71 (d, 8 H, *J* = 7.0 Hz), 7.63-7.59 (m, 12 H), 6.85 (d, 2 H, *J* = 13.0 Hz); λ_{max} (CH_2Cl_2) = 806 nm (ε = 2.5 × 10^5 $M^{-1}cm^{-1}$); LRMS (ESI) *m/z* 631.2 (calcd for $C_{37}H_{27}{}^{80}Se_2^+$: 631.0). **Anal.** Calcd for $C_{37}H_{27}Se_2 \cdot PF_6$: C, 57.38; H, 3.51; F, 14.72. Found: C, 57.34; H, 3.48; F, 14.76.

Synthesis of 4-(3-(2,6-diphenyl-4H-thiopyran-4-ylidene)prop-1-enyl)-2,6-diphenylselenopyrylium hexafluorophosphate (10)[S4]

4-Methyl-2,6-di(phenyl)thiopyrylium hexafluorophosphate (0.128 g, 0.312 mmol), 4-(2,6-diphenyl-4H-selenopyran-4ylidene)acetaldehyde (0.157 g, 0.344 mmol) and Ac_2O (2.0 mL) were combined in a round bottom flask and heated at 105 °C for 10 min. The reaction was cooled to ambient temperature, CH_3CN (2.0 mL) was added and ether was used to precipitate product from solution to yield 0.196 g (86%) of a copper-bronze solid: ^{1}H NMR [500 MHz, CD_2Cl_2] δ 8.54 (t, 1 H, *J* = 13.0 Hz), 8.20-7.80 (br s, 4 H), 7.78 (d, 4 H, *J* = 8.0 Hz), 7.70 (d, 4 H, *J* = 7.5 Hz), 7.66-7.58 (m, 12 H), 6.78 (d, 2 H, *J* = 13.5 Hz); λ_{max} (CH_2Cl_2) = 784 nm (ε 2.0 × 10^5 $M^{-1}cm^{-1}$); LRMS (ESI) *m/z* 583.3 (calcd for $C_{37}H_{27}S^{80}Se^+$: 583.1). **Anal.** Calcd for $C_{39}H_{34}O_3Se_2 \cdot PF_6$: C, 61.08; H, 3.74. Found: C, 61.10; H, 3.68.

Synthesis of 4-(3-(2,6-dithiophen-2 -yl-4H-thiopyran-4-ylidene)prop-1-enyl)-2,6-diphenylselenopyrylium hexafluorophosphate (11)

4-Methyl-2,6-di(phenyl)selenopyrylium hexafluorophosphate (0.102 g, 0.225 mmol), 4-(2,6-(thiophene-2-yl)-4H-thiopyran-4ylidene)acetaldehyde (75.0 mg, 0.248 mmol) and Ac_2O (3.0 mL) were combined in a round bottom flask and heated at 105 °C for 5 min. The reaction was cooled to ambient temperature, precipitated with ether, and the collected solid recrystallized from CH_3CN/ether to yield 0.145 g (87%) of a bronze solid, mp 229-231 °C: ^{1}H NMR [500 MHz, CD_2Cl_2] δ 8.46 (t, 1 H, *J* = 13.0 Hz), 7.71-7.58 (m, 18 H), 7.26 (t, 2 H, *J* = 4.0 Hz), 6.77 (d, 1 H, *J* = 13.0 Hz), 6.70 (d, 1 H, *J* = 14.0 Hz); λ_{max} (CH_2Cl_2) = 810 nm (ε 2.5 × 10^5 $M^{-1}cm^{-1}$); HRMS (ESI, HRDFMagSec) *m/z* 595.0125 (calcd for $C_{33}H_{23}S_3{}^{80}Se^+$: 595.0122). **Anal.** Calcd for $C_{33}H_{23}S_3Se \cdot PF_6$: C, 53.59; H, 3.13; F, 15.41. Found: C, 53.79; H, 3.13; F, 15.19.

Synthesis of 4-(3-(2,6-dithiophen-2-yl-4H-thiopyran-4-ylidene)prop-1-enyl)-2,6-diphenylthiopyrylium hexafluorophosphate (12)

4-Methyl-2,6-diphenylthiopyrylium hexafluorophosphate (30.0 mg, 73.0 μmol), 4-(2,6-(thiophene-2-yl)-4H-thioopyran-4ylidene)acetaldehyde (24.4 mg, 81.0 μmol) and Ac_2O (1.0 mL) were combined in a round bottom flask and heated at 105 °C for 5 min. The reaction was cooled to ambient temperature, CH_3CN (4.0 mL) was added and ether was used to precipitate product from solution to yield 45.0 mg (88%) of a bronze solid, mp > 260 °C: ^1H NMR [500 MHz, CD_2Cl_2] δ 8.44 (t, 1 H, J = 13.0 Hz), 8.40-7.80 (br s, 4 H), 7.78 (d, 4 H, J = 7.0 Hz), 7.67-7.59 (m, 10 H), 7.24 (t, 2 H, J = 4.5 Hz), 6.71 (d, 1 H, J = 13.0 Hz), 6.63 (d, 1 H, J = 13.5 Hz); λ_{max} (CH_2Cl_2) = 789 nm (ε 2.2 × 10^5 $M^{-1}cm^{-1}$); HRMS (ESI, HRDFMagSec) *m/z* 547.0674 (calcd for $C_{33}H_{23}S_4^+$: 547.0677). **Anal.** Calcd for $C_{33}H_{23}S_4 \cdot PF_6$: C, 57.21; H, 3.35. Found: C, 56.97; H, 3.36.

Synthesis of 4-(3-(2,6-dithiophen-2-yl-4H-thiopyran-4-ylidene)prop-1-enyl)-(2,6-dithiophen-2-yl)thiopyrylium hexafluorophosphate (13)

4-Methyl-2,6-di(thiophen-2-yl)thiopyrylium hexafluorophosphate (11.0 mg, 26.2 μmol), 4-(2,6-(thiophene-2-yl)-4H-thioopyran-4ylidene)acetaldehyde (9.5 mg, 31.4 μmol) and Ac_2O (1.0 mL) were combined in a round bottom flask and heated at 105 °C for 5 min. The reaction was cooled to ambient temperature, CH_2Cl_2 (2.0 mL) was added and ether was used to precipitate product from solution to yield 17.8 mg (94%) of a bronze solid, mp > 260 °C: ^1H NMR [500 MHz, CD_3CN] δ 8.32 (t, 1 H, J = 13.5 Hz), 7.68 (d, 2 H, J = 4 Hz), 7.56 (br s, 4 H) 7.14 (t, 4 H, J = 4.5 Hz), 6.48 (d, 2 H, J = 13.0 Hz); λ_{max} (CH_2Cl_2) = 813 nm (ε 2.8 × 10^5 $M^{-1}cm^{-1}$); HRMS (ESI, HRDFMagSec) *m/z* 558.9805 (calcd for $C_{29}H_{19}S_6^+$: 558.9806). **Anal.** Calcd for $C_{29}H_{19}S_6 \cdot PF_6$: C, 49.42; H, 2.72. Found: C, 49.19; H, 2.79.

Synthesis of 4-(3-(2,6-di(selenophen-2-yl)-4*H*-thiopyran-4-ylidene)prop-1-en-1-yl)-2,6-di(selenophen-2-yl)thiopyrylium hexafluorophosphate (14)

4-Methyl-2,6-di(selenophen-2-yl)thiopyrylium hexafluorophosphate (47.1 mg, 91.4 μmol), 2-(2,6-di(selenophen-2-yl)-4*H*-thiopyran-4-ylidene)acetaldehyde (47.1 mg, 0.101 mmol) and Ac_2O (2.0 mL) were heated for 5 min at 105 °C prior to cooling to ambient temperature, diluting with

CH_3CN (3.0 mL) and adding ether to precipitate the product to yield 71.0 mg (87%) of a copper bronze solid, mp 249-251 °C: ^{1}H NMR [500 MHz, CD_3CN] δ 8.51-8.46 (m, 5 H), 7.88 (d, 4 H, *J* = 3.0 Hz), 7.71 (br s, 4 H), 7.46 (t, 4 H, *J* = 4.5 Hz), 6.62 (d, 2 H, *J* = 13.0 Hz); λ_{max} (CH_2Cl_2) = 826 nm (ε 2.3 × 10^5 $M^{-1}cm^{-1}$); 750 nm (ε 5.2 × 10^4 $M^{-1}cm^{-1}$); 490 nm (ε 2.8 × 10^4 $M^{-1}cm^{-1}$); HRMS (ESI, HRDFMagSec) *m/z* 750.7560 (calcd for $C_{29}H_{19}S_2{}^{80}Se_4{}^+$: 750.7584). **Anal.** Calcd for $C_{29}H_{19}S_2Se_4{\cdot}PF_6$: C, 39.03; H, 2.15. Found: C, 39.28; H, 2.19.

Synthesis of HGNs

The HGN synthesis was carried out under inert conditions using a standard Schlenk line to prevent the cobalt nanoparticles from prematurely oxidising. The method described was modified slightly from previous reports.[S6, S7] In a typical synthesis, cobalt chloride hexahydrate (100 µL, 0.4M; Fisher Scientific, 99.99%) and trisodium citrate dihydrate (550 µL, 0.1 M; Sigma-Aldrich, >99%) were added into deionised water (100 mL) and degassed several times (10 mins vacuum and 15 mins argon). Sodium borohydride (1 mL, 0.1 M; Fisher Scientific, 99%) was injected into the solution and allowed to react for a further 20 minutes (under constant argon flow) until hydrogen evolution ceased, indicating complete hydrolysis of the reductant. The solution was degassed again (8 min vacuum and 10 min argon) before chloroauric acid trihydrate (33 mL, 248 µM; Fisher Scientific, ACS reagent grade) was injected. This mixture was allowed to react for an additional 10 minutes under argon with vigorous stirring. Before being exposed to air, an obvious colour change from brown to green was observed. Finally, trisodium citrate (500 µL, 0.1 M) was added to stabilise the hollow gold nanoshell solution. Post synthesis, the HGN solution was concentrated through centrifugation (5000 x rpm) and the precipitate was re-dispersed in trisodium citrate solution (2mM) to give a final concentration of 2.97 nM. The HGNs prepared in this manner had a surface plasmon resonance at 720 nm, figure S2.

Characterisation of HGNs

Investigation into the SERS properties of the HGNs were carried out by mixing concentrated HGN solution (270 µL) with Raman reporter solution; namely dyes 1-14, BPE and AZPY (40 µL, 10 µM; synthesised in-house or purchased from Sigma-Aldrich) and potassium chloride (300 µL, 30 mM; Sigma-Aldrich). The Raman measurements were performed using a SnRI portable

Raman spectrometer and a laser excitation wavelength of 1280 nm. All the measurements had a 3-7 second acquisition time and a laser power operating at 100 mW. Each sample was prepared in triplicate and 5 scans of each replicate were recorded. Furthermore, all the Raman spectra have been background corrected. For the SERS particle dilution studies, the optimum conditions were used (as stated above) and deionised water was added to obtain subsequent concentrations, over the concentration range 1.93 nM to 6 pM. All other experimental conditions were kept the same as those stated above. The limit of detection (LOD) was calculated to be 3 times the standard deviation of the blank, divided by the gradient of the straight line. Error bars represent one standard deviation resulting from 3 replicate samples and 5 scans of each, see table S1 for the LOD values for dyes 1-14 and figure S7 for associated LOD plots with error bars for dyes 1-13. The extinction spectrum for the HGNs with SPR at 720 nm was acquired using a Varian Cary 300 Bio-UV-Visible Spectrophotometer over the wavelength range 400-900 nm. While the extinction spectra showing the effects of aggregation were acquired using a Perkin Elmer Lambda 35 UV-Vis Spectrometer with an extended wavelength range up to 1100 nm.

Chemometrics

Principal component analysis (PCA) was performed to assess the reproducibility and separation of individual features within the data set using Matlab software version R2012a (The MathWorks, Natick, MA, USA). PCA reduces the dimensionality of the SERS data, making it easier to identify any variations in the spectra.[S8, S9] PCA was carried out on 14 data sets, consisting of the spectra obtained from each individual dye experiment.

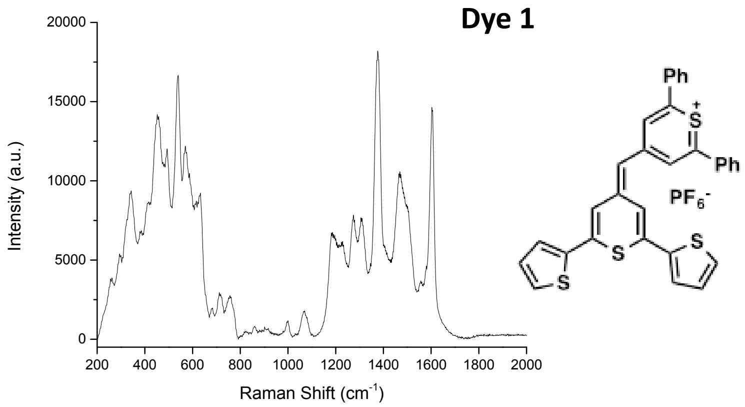
Dye 1
Intensity (a.u.)
Raman Shift (cm^{-1})
Ph
S
Ph
PF$_6^-$
S
S
S

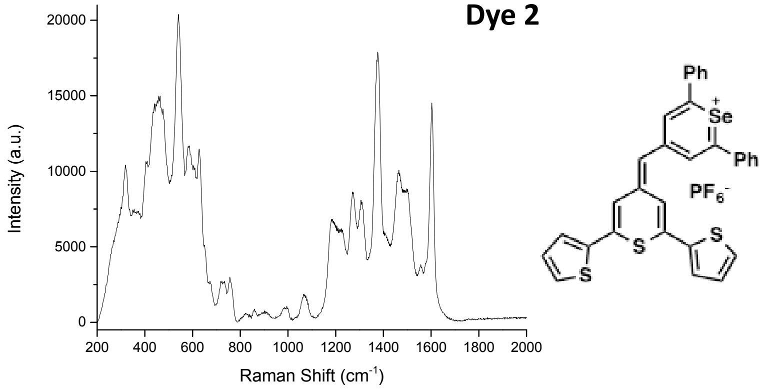
Dye 2
Intensity (a.u.)
Raman Shift (cm^{-1})
Ph
Se
Ph
PF$_6^-$
S
S
S

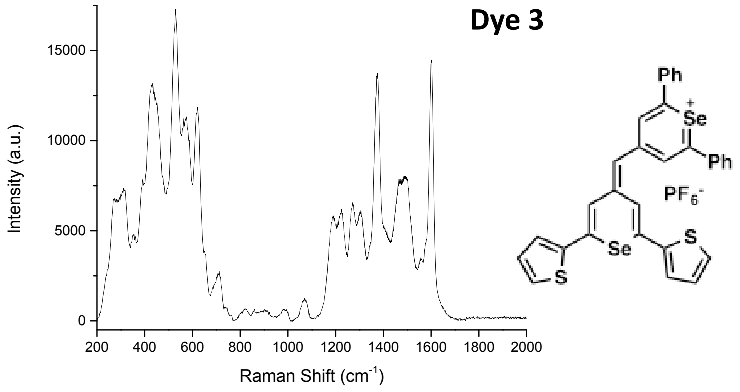
Dye 3
Intensity (a.u.)
Raman Shift (cm^{-1})
Ph
Se
Ph
PF$_6^-$
Se
S
S

Dye 4

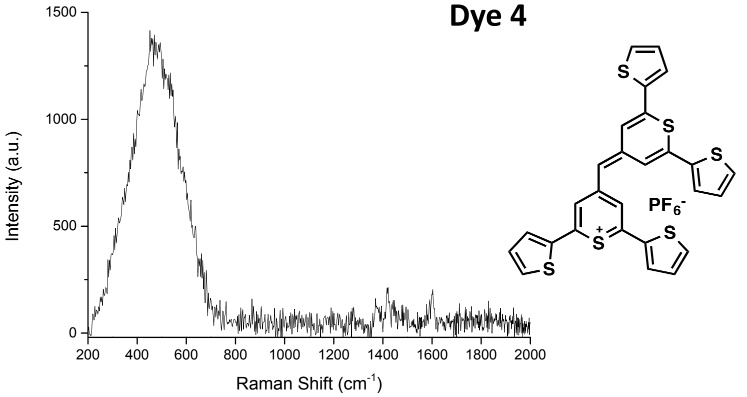

Dye 5

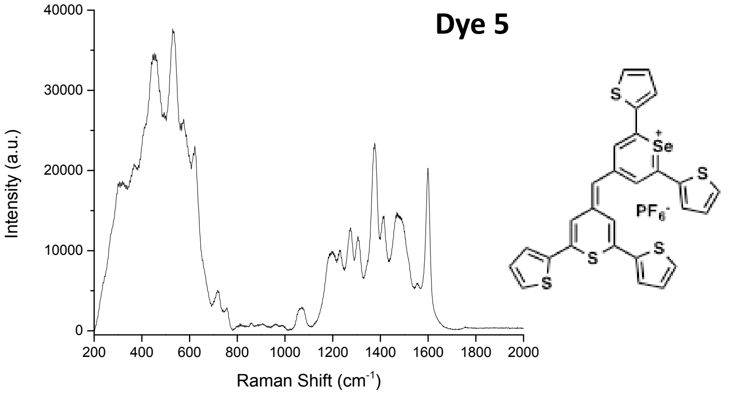

Dye 6

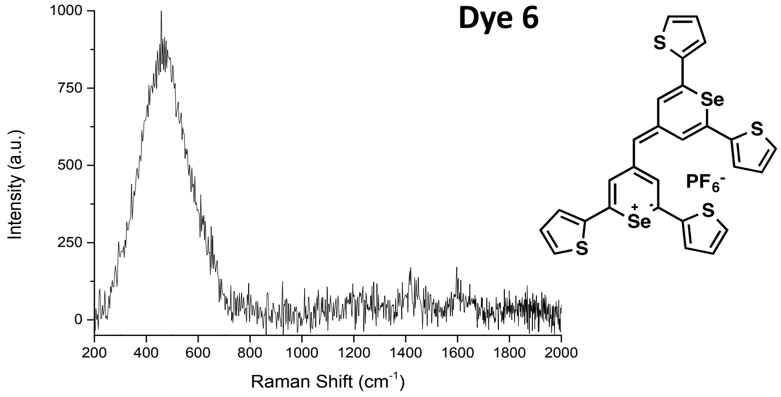

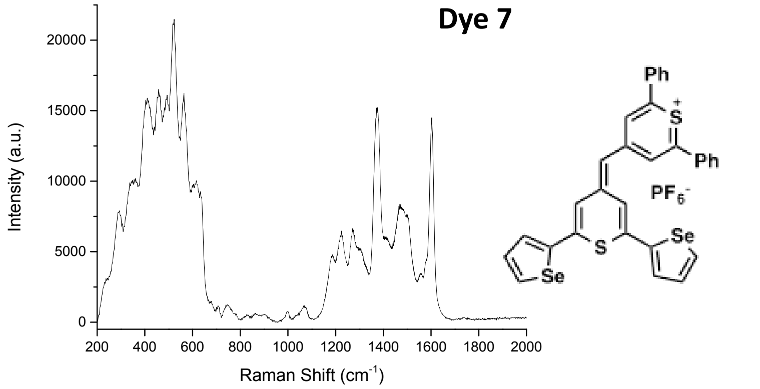
Dye 7
Intensity (a.u.)
Raman Shift (cm-1)
Ph
S
Ph
PF6-
Se
S
Se

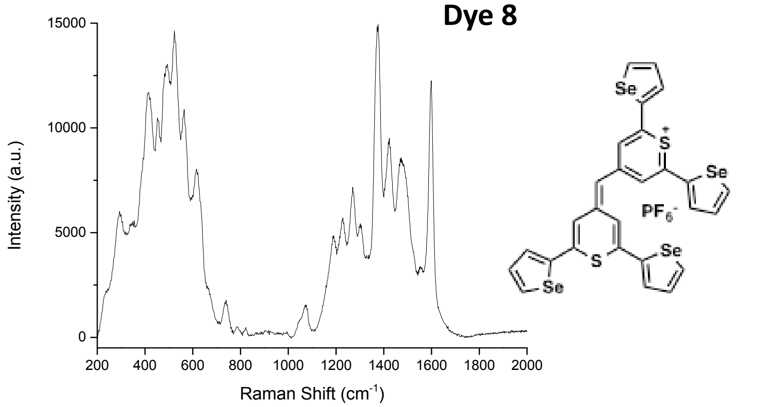
Dye 8
Intensity (a.u.)
Raman Shift (cm-1)
Se
S
Se
PF6-
Se
S
Se

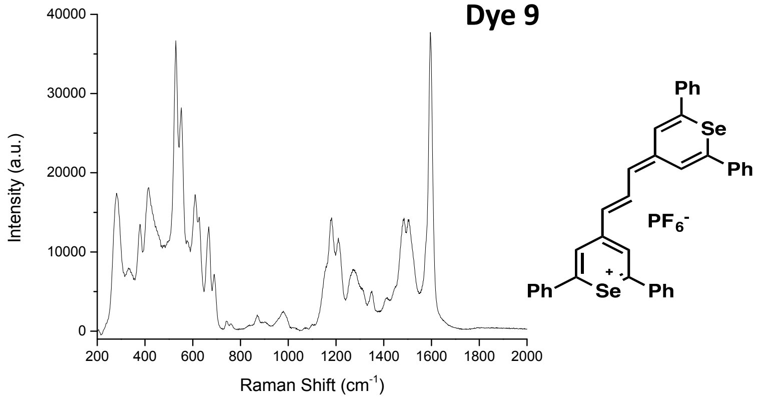
Dye 9
Intensity (a.u.)
Raman Shift (cm-1)
Ph
Se
Ph
PF6-
Ph
Se
Ph

Dye 10

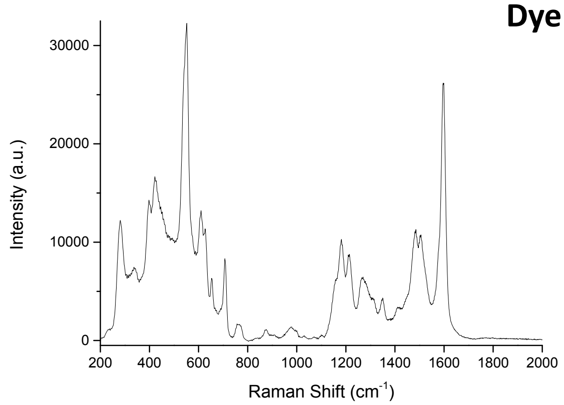

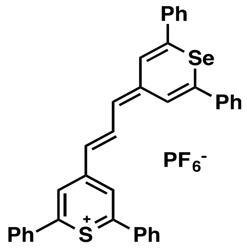

Dye 11

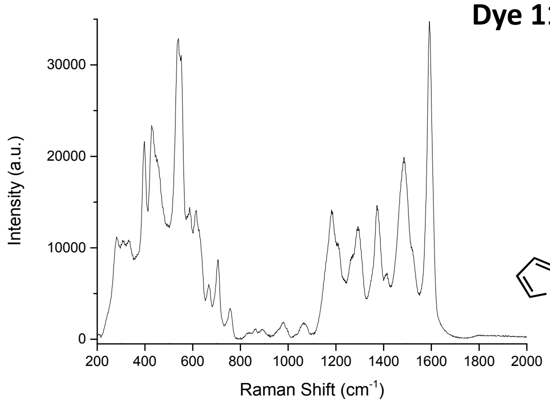

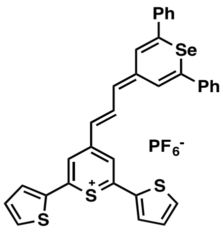

Dye 12

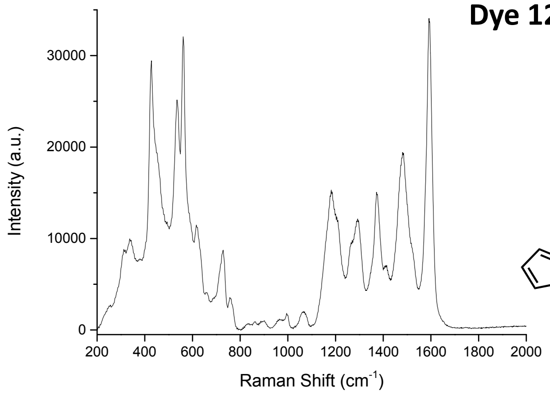

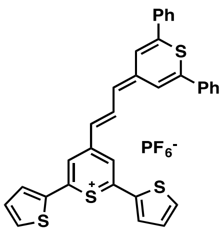

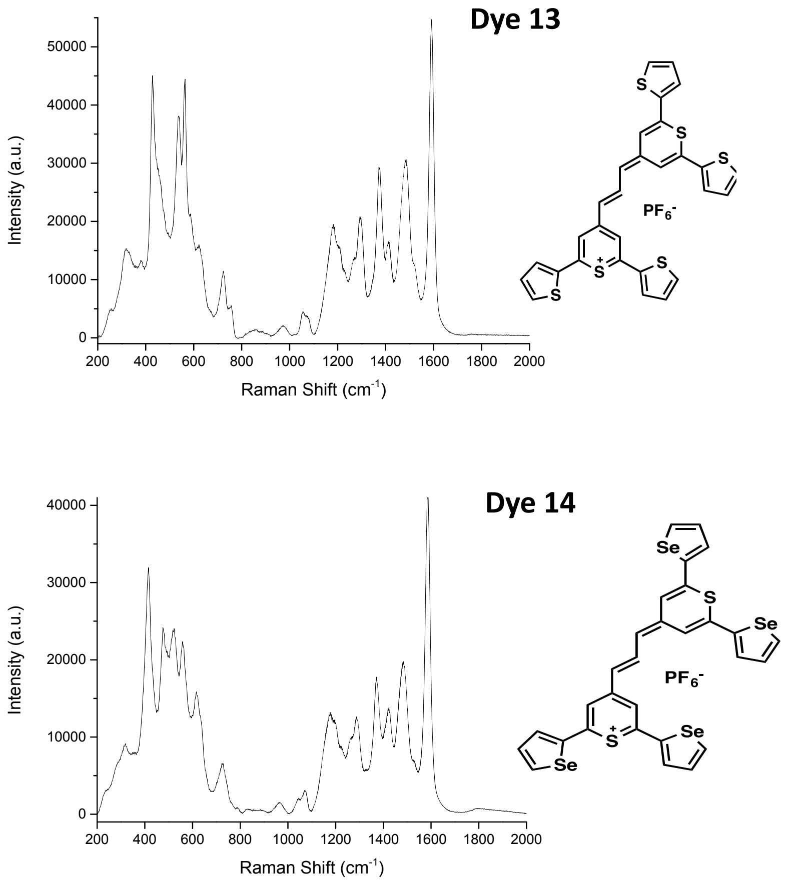

Figure S1. SERS spectra and structures of dyes 1-14. The dyes with a concentration of 10 µM were analysed with HGNs (SPR recorded at 720 nm) and KCl. A laser excitation of 1280 nm and an exposure time of 7 seconds were employed in this analysis, with the exception of dye 14 which had an acquisition time of 3 seconds. All spectra have been background corrected.

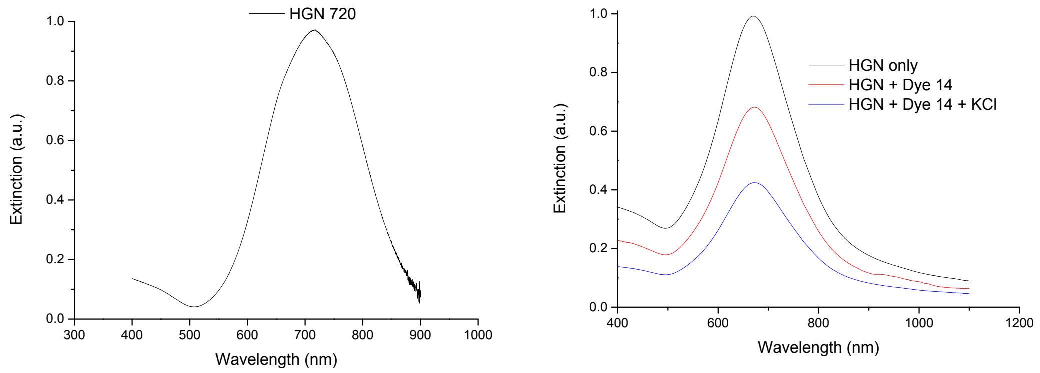

Figure S2. a) Extinction spectrum showing the SPR of the HGNs at 720 nm. b) Extended extinction spectra showing that the SPR does not shift upon aggregation. The black line highlights the HGN only spectrum; red line is HGN plus dye 14 (10 µM) and the blue line is HGN plus dye 14 and KCl salt (30 mM).

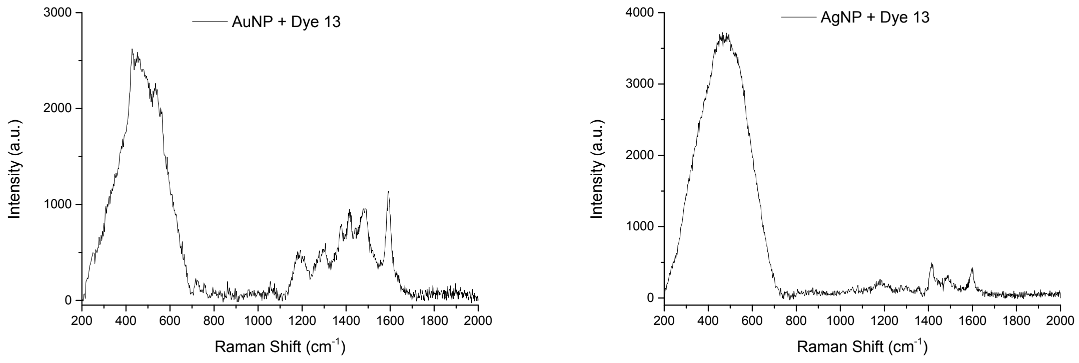

Figure S3. SERS spectra for dye **13** with gold and silver nanoparticles and aggregated with KCl. A laser excitation of 1280 nm and an exposure time of 7 seconds were employed in this analysis. All spectra have been background corrected.

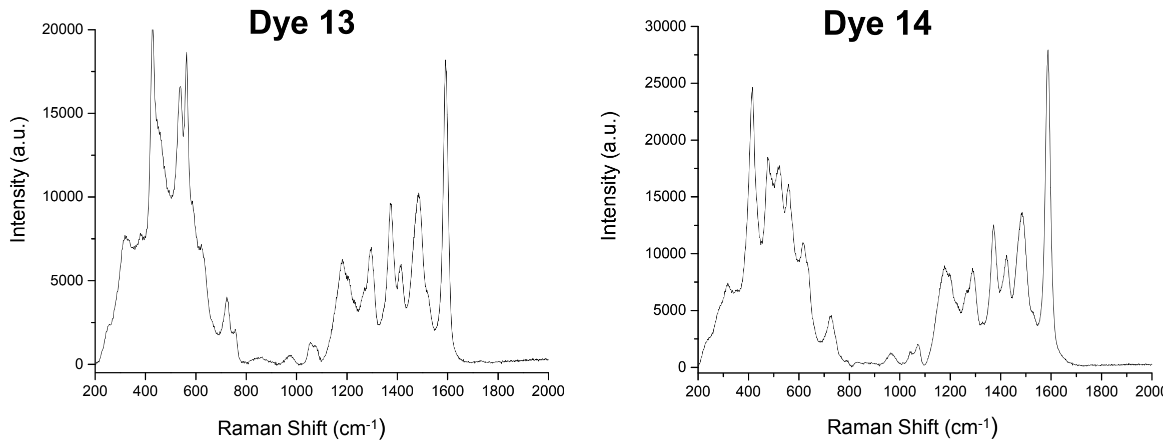

Figure S4. Unaggregated SERS spectra of dyes **13** and **14** analyzed with HGNs (SPR recorded at 720 nm) and deionised water. A laser excitation of 1280 nm and an exposure time of 7 seconds were employed in this analysis. All spectra have been background corrected.

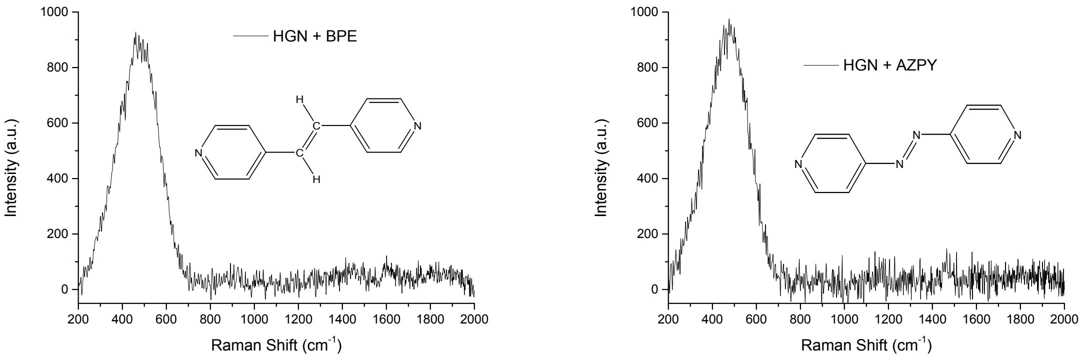

Figure S5. SERS spectra and structures of commercial dyes BPE and AZPY with HGNs (SPR recorded at 720 nm) and KCl. A laser excitation of 1280 nm and an exposure time of 7 seconds were employed in this analysis. All spectra have been background corrected.

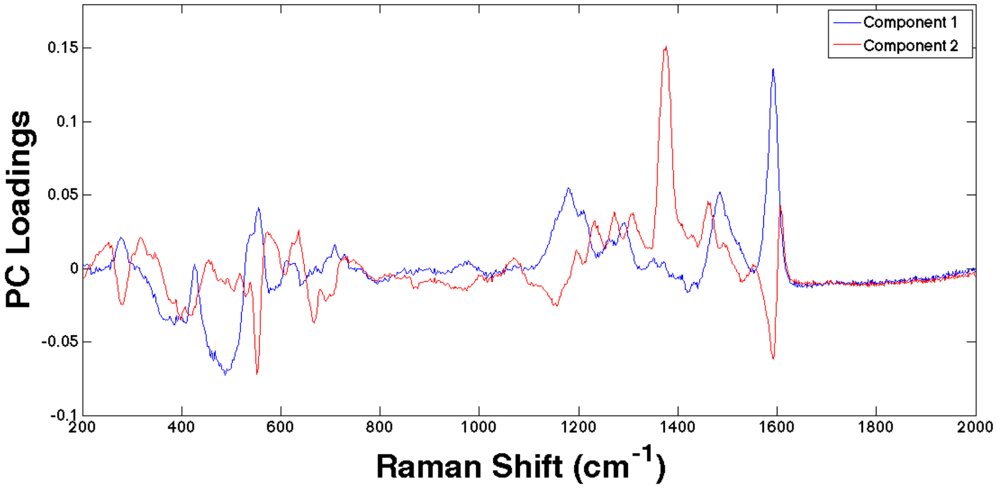

Figure S6. PC Loadings plots showing the Raman peaks that are responsible for the classification of the dyes across components 1 and 2.

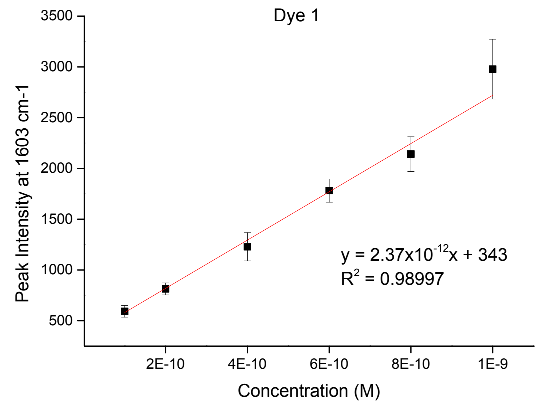
Dye 1
Peak Intensity at 1603 cm-1
Concentration (M)
y = 2.37x10^-12x + 343
R^2 = 0.98997

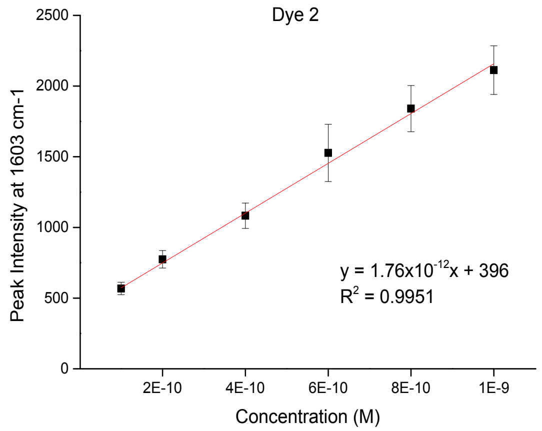
Dye 2
Peak Intensity at 1603 cm-1
Concentration (M)
y = 1.76x10^-12x + 396
R^2 = 0.9951

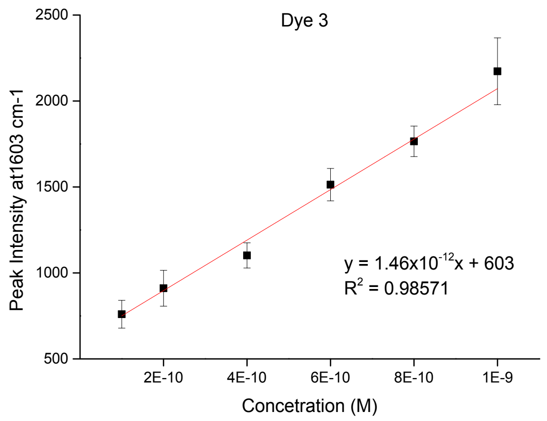
Dye 3
Peak Intensity at1603 cm-1
Concetration (M)
y = 1.46x10^-12x + 603
R^2 = 0.98571

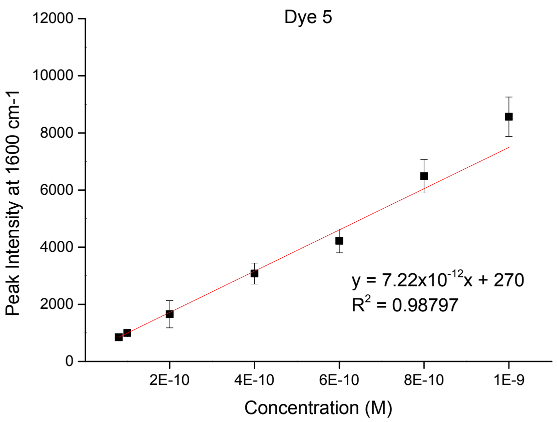
Dye 5
Peak Intensity at 1600 cm-1
Concentration (M)
y = 7.22x10^-12x + 270
R^2 = 0.98797

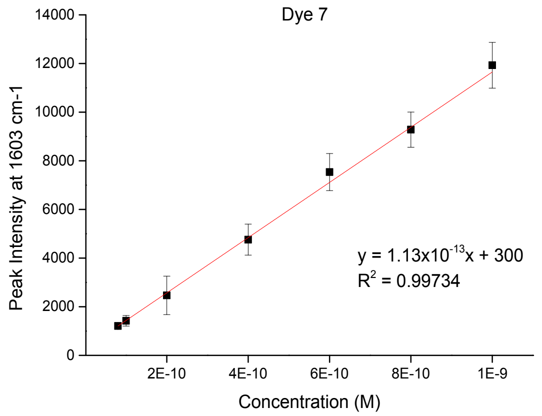
Dye 7
Peak Intensity at 1603 cm-1
Concentration (M)
y = 1.13x10^-13x + 300
R^2 = 0.99734

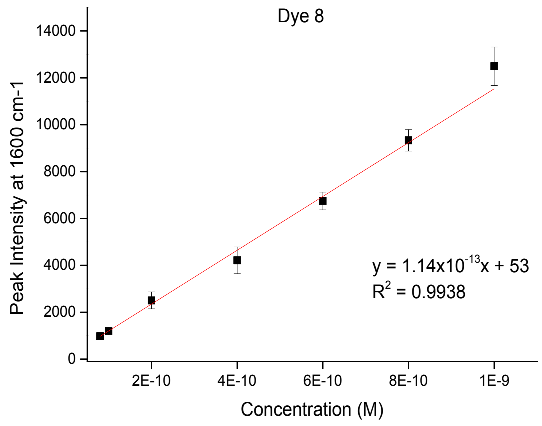
Dye 8
Peak Intensity at 1600 cm-1
Concentration (M)
y = 1.14x10^-13x + 53
R^2 = 0.9938

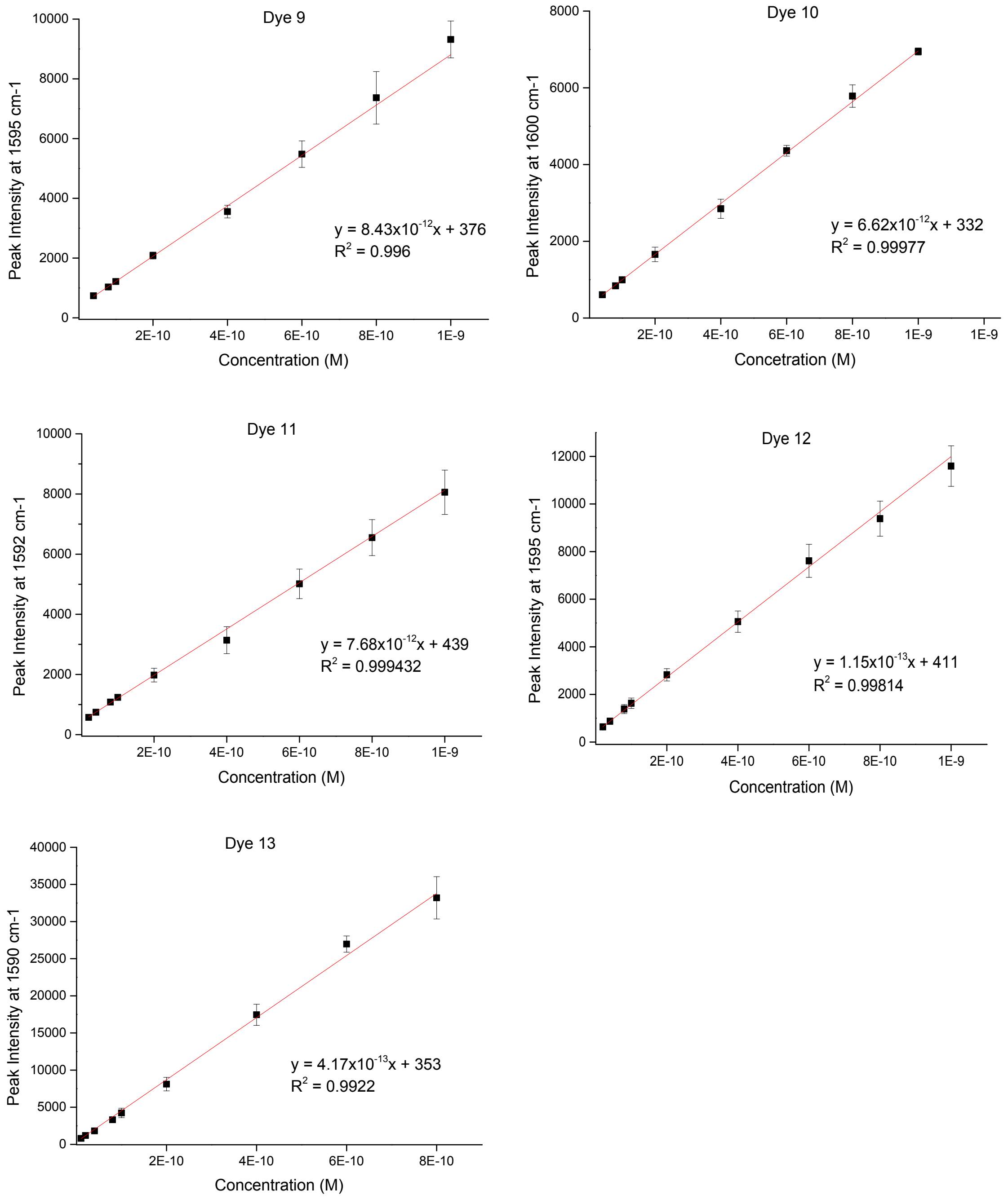

Figure S7. SERS particle dilution studies for dye **1-13** with HGNs and KCl over the concentration range 1.93 nM to 6 pM. Dyes **4 and 6** were not analysed due to their weak SERS response at the optimum concentration. A laser excitation of 1280 nm and an exposure time of 7 seconds were employed in this analysis. Error bars represent one standard deviation resulting from 3 replicate samples and 5 scans of each.

References

S1. K. Leonard, M. Nelen, M. Raghu and M. R. Detty, *J. Heterocycl. Chem.*, 1999, **36**, 707-717.

S2. M. A. Kudinova, A. D. Kachkovski, V. V. Kurdyukov and A. I. Tolmachev, *Dyes Pigm.*, 2000, **45**, 1-7.

S3. M. R. Detty, J. M. McKelvey and H. R. Luss, *Organometallics*, 1988, **7**, 1131-1147.

S4. M. R. Detty and B. J. Murray, *J. Org. Chem.*, 1982, **47**, 5235-5239.

S5. S. Kim, T. Y. Ohulchanskyy, A. Baev and P. N. Prasad, *J. Mater. Chem.*, 2009, **19**, 3181-3188.

S6. A. M. Schwartzberg, T. Y. Olson, C. E. Talley and J. Z. Zhang, *The Journal of Physical Chemistry B*, 2006, **110**, 19935-19944.

S7. H.-n. Xie, I. A. Larmour, Y.-C. Chen, A. W. Wark, V. Tileli, D. W. McComb, K. Faulds and D. Graham, *Nanoscale*, 2013, **5**, 765-771.

S8. D.-H. Kim, R. M. Jarvis, Y. Xu, A. W. Oliver, J. W. Allwood, L. Hampson, I. N. Hampson and R. Goodacre, *Analyst*, 2010, **135**, 1235-1244.

S9. S. Mabbott, A. Eckmann, C. Casiraghi and R. Goodacre, *Analyst*, 2013, **138**, 118-122.

Crystallographic details for the X-ray crystal structure of dye 14

X-ray diffraction data was collected on a Bruker SMART APEX2 CCD diffractometer installed at a rotating anode source (Mo Kα radiation, λ=0.71073 Å), and equipped with an Oxford Cryosystems (Cryostream700) nitrogen gas-flow apparatus. The crystal was kept at 90 K during data collection. The data was collected by the rotation method with a 0.5° frame-width (ω scan) and a 20 second exposure per frame. Five sets of data (360 frames in each set) were collected, nominally covering complete reciprocal space.

Using Olex2[1], the structure was solved with the XS[2] structure solution program using Direct Methods and refined with the XL[2] refinement package using Least Squares minimisation. All non-hydrogen atoms were refined anisotropically. The asymmetric unit consists of one unique dye **14** molecule, one PF_6^- counter-ion, and three acetonitrile solvent molecules. Over the course of approximately 2-3 weeks, the single crystals showed marked degradation, presumably from the loss of solvent molecules.

Three of the selenophene rings showed evidence of minor crystallographic disorder. For all three disordered rings, the minor species was restrained to be planar with bond lengths similar to the major species by using FLAT and SADI commands. In a pair-wise manner, thermal ellipsoids of the disordered atoms were restrained to be same using EADP. The final refined occupancies for the minor species of the three rings were calculated to be 0.0435(17), 0.0379(17), and 0.0745(18).

The coplanarity of the aromatic groups in dye **14** was assessed by calculating the angle between the mean plane of the aromatic groups. The mean plane for each group was calculated using only the non-hydrogen atoms of a given group (MPLN command in Olex2). The angle between sets of two mean planes was then calculated using the ESD command in Olex2. The results are shown in Figure S8.

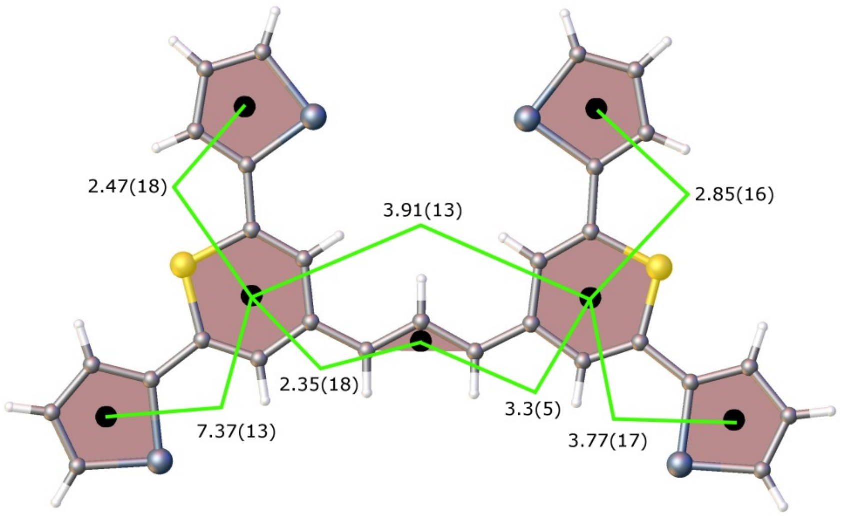

Figure S8. Diagram indicating the angle (in degrees given with standard deviation) between the calculated mean planes of the aromatic groups of dye **14** using single crystal X-ray diffraction data. The black dots indicate the centroid of the mean plane for each individual aromatic group.

Table S1 Crystal data and structure refinement for Dye 14.	
Identification code	Dye 14
Empirical formula	$C_{35}H_{28}F_6N_3PS_2Se_4$
Formula weight	1015.53
Temperature/K	90
Crystal system	monoclinic
Space group	$P2_1/n$
a/Å	19.6176(12)
b/Å	7.5992(5)
c/Å	25.2231(15)
α/°	90
β/°	93.7263(19)
γ/°	90
Volume/Å^3	3752.3(4)
Z	4
ρ_{calc}g/cm^3	1.798
μ/mm^{-1}	4.126
F(000)	1984.0
Crystal size/mm^3	0.08 × 0.02 × 0.02
Radiation	MoKα (λ = 0.71073)
2Θ range for data collection/°	3.236 to 54.302
Index ranges	$-25 \leq h \leq 25, -9 \leq k \leq 9, -32 \leq l \leq 32$
Reflections collected	69197
Independent reflections	8308 [R_{int} = 0.1034, R_{sigma} = 0.0651]
Data/restraints/parameters	8308/22/502
Goodness-of-fit on F^2	1.087
Final R indexes [I>=2σ (I)]	R_1 = 0.0513, wR_2 = 0.0967
Final R indexes [all data]	R_1 = 0.0911, wR_2 = 0.1075
Largest diff. peak/hole / e Å^{-3}	0.55/-0.64

Table S2 Fractional Atomic Coordinates (×10^4) and Equivalent Isotropic Displacement Parameters (Å^2×10^3) for Dye 14. U_{eq} is defined as 1/3 of of the trace of the orthogonalised U_{IJ} tensor.

**Atom**	***x***	***y***	***z***	**U(eq)**

Se1	2296.0(2)	6056.5(7)	6420.2(2)	23.25(13)
S1	319.3(6)	4041.9(17)	6967.8(5)	19.3(3)
S2	1528.6(6)	10994.8(17)	3454.5(5)	19.7(3)
P1	4294.9(7)	7944(2)	6141.9(6)	28.9(3)
F1	4606.7(19)	7478(5)	5592.6(13)	51.2(10)
F2	4393(2)	9976(5)	6031.5(13)	48(1)
F3	3972.5(18)	8358(5)	6688.7(14)	48.6(10)
F4	4185(2)	5865(5)	6249.4(15)	60.8(11)
F5	3557.5(17)	7986(5)	5860.6(15)	51.3(10)
F6	5019.7(18)	7782(6)	6436.5(14)	61.5(12)
N1	1447(3)	10053(8)	5926(3)	56.2(16)
N2	1765(3)	5100(8)	4965(2)	49.8(15)
N3	3522(3)	5791(8)	3653(2)	54.6(16)
C1	2886(3)	5342(7)	6977(2)	23.2(11)
C2	2568(3)	4459(7)	7351(2)	27.5(13)
C3	1847(3)	4262(7)	7251(2)	29.5(13)
C4	1596(2)	5005(6)	6775.0(18)	17(1)
C5	889(2)	5026(6)	6557.7(18)	17.1(10)
C6	690(2)	5784(6)	6090.7(18)	17(1)
C7	10(2)	5915(6)	5863.9(18)	15.9(10)
C8	-533(2)	5179(6)	6147.8(18)	15.9(10)
C9	-468(2)	4373(6)	6624.5(18)	15.7(10)
C10	-1047(2)	3716(6)	6902.8(19)	19.3(11)
C14	-160(2)	6739(6)	5373.7(18)	15.9(10)
C15	305(2)	7561(6)	5063.7(17)	15.5(10)
C16	121(2)	8332(6)	4575.1(19)	18.5(11)
C20	340(2)	11374(6)	2881.8(19)	20.5(11)
C21	650(2)	10686(6)	3380.0(18)	16.2(10)
C22	297(2)	9889(6)	3752.9(18)	16.6(10)
C23	574(2)	9191(6)	4248.7(18)	16.2(10)
C24	1286(2)	9388(6)	4390.5(19)	18(1)
C25	1743(2)	10141(6)	4083.8(19)	17.8(10)
C26	2470(3)	10274(7)	4238.4(19)	21.1(11)
C30	1958(3)	10553(7)	6089(3)	35.4(14)
C31	2633(3)	11159(8)	6284(2)	37.7(14)
C32	2331(3)	4828(8)	5000(2)	31.1(13)
C33	3065(3)	4524(8)	5058(3)	41.1(15)
C34	3998(3)	6079(8)	3922(2)	37.0(14)
C35	4614(3)	6427(9)	4273(2)	46.6(17)
Se2	-1944.4(3)	4039.1(8)	6628.4(2)	22.34(17)

Se3	-574.4(3)	11004.8(8)	2676.2(2)	24.77(17)
Se4	2837.3(3)	9372.8(8)	4876.7(2)	26.03(19)
C11	-1021(4)	2837(10)	7377(3)	24.1(17)
C12	-1667(3)	2354(7)	7551(2)	26.2(13)
C13	-2211(3)	2862(7)	7222(2)	26.4(13)
C17	-436(3)	12360(8)	2082(2)	28.7(14)
C18	216(3)	12928(7)	2074(2)	25.0(13)
C19	643(3)	12373(8)	2508(2)	21.1(14)
C27	2971(4)	11109(11)	3968(3)	39(2)
C28	3634(3)	10968(9)	4213(3)	33.9(15)
C29	3683(3)	10164(9)	4689(3)	27.9(16)
Se2A	-1078(10)	2700(30)	7540(6)	22.34(17)
Se3A	762(9)	12140(30)	2340(6)	24.77(17)
Se4A	3099(5)	10905(14)	3808(4)	26.03(19)
C11A	-1670(20)	4110(90)	6660(30)	24.1(17)
C12A	-2207(15)	3650(140)	6980(40)	26.2(13)
C13A	-2016(16)	2930(130)	7460(40)	26.4(13)
C17A	-80(30)	12490(140)	1990(50)	28.7(14)
C18A	-620(70)	12060(160)	2270(40)	25.0(13)
C19A	-352(16)	11430(110)	2760(50)	21.1(14)
C27A	2800(40)	9800(70)	4719(17)	39(2)
C28A	3520(40)	9900(120)	4790(40)	33.9(15)
C29A	3770(40)	10430(90)	4340(30)	27.9(16)

Table S3 Anisotropic Displacement Parameters ($Å^2×10^3$) for Dye 14. The Anisotropic displacement factor exponent takes the form: $-2\pi^2[h^2a^{*2}U_{11}+2hka^*b^*U_{12}+…]$.

Atom	U_{11}	U_{22}	U_{33}	U_{23}	U_{13}	U_{12}
Se1	17.8(3)	26.0(3)	25.7(3)	7.2(2)	-0.5(2)	-2.5(2)
S1	19.8(6)	19.2(6)	19.1(6)	4.7(5)	1.9(5)	-2.1(5)
S2	20.9(6)	21.3(6)	17.0(6)	4.7(5)	1.6(5)	0.2(6)
P1	24.6(8)	36.8(9)	25.3(8)	-2.7(7)	3.1(6)	-2.6(7)
F1	67(3)	54(2)	34(2)	0.2(18)	11.2(18)	14(2)
F2	72(3)	37(2)	35(2)	-1.8(17)	8.2(18)	-19.6(19)
F3	57(2)	49(2)	42(2)	-5.7(18)	17.5(18)	-3.6(19)
F4	78(3)	47(2)	60(3)	6(2)	23(2)	10(2)

F5	38(2)	51(2)	64(3)	-11(2)	-6.4(18)	0.3(18)
F6	35(2)	109(4)	40(2)	10(2)	-3.4(16)	3(2)
N1	33(3)	46(4)	88(5)	19(3)	-8(3)	5(3)
N2	35(3)	54(4)	60(4)	-5(3)	-1(3)	-7(3)
N3	39(3)	77(5)	48(3)	-6(3)	5(3)	-9(3)
C1	17(3)	26(3)	26(3)	-2(2)	-2(2)	4(2)
C2	19(3)	36(3)	26(3)	5(2)	-3(2)	1(2)
C3	21(3)	35(3)	32(3)	8(3)	-3(2)	4(2)
C4	20(3)	15(2)	16(2)	0(2)	-1(2)	1(2)
C5	20(3)	14(2)	18(3)	1(2)	4(2)	1(2)
C6	16(2)	17(3)	19(2)	-1(2)	4.2(19)	0(2)
C7	15(2)	15(2)	18(2)	-5(2)	3.2(18)	-1(2)
C8	14(2)	15(2)	19(3)	-1(2)	1.5(19)	2(2)
C9	17(2)	13(2)	18(2)	0(2)	5.6(19)	-3.3(19)
C10	25(3)	13(2)	21(3)	-7(2)	9(2)	-2(2)
C14	17(2)	16(2)	14(2)	-4(2)	-2.0(19)	0(2)
C15	17(2)	17(2)	12(2)	-4(2)	0.7(19)	2(2)
C16	20(3)	16(2)	20(3)	-4(2)	5(2)	-1(2)
C20	26(3)	17(3)	18(3)	-3(2)	0(2)	7(2)
C21	21(2)	14(2)	13(2)	-3.7(19)	-2.0(19)	4(2)
C22	14(2)	15(2)	20(3)	-5(2)	-1.4(19)	0(2)
C23	18(2)	15(2)	16(2)	-3(2)	4.0(18)	4(2)
C24	19(2)	21(3)	15(2)	3(2)	1.0(19)	-1(2)
C25	23(3)	12(2)	19(3)	-2(2)	-1(2)	3(2)
C26	21(3)	20(3)	23(3)	3(2)	2(2)	-2(2)
C30	30(3)	25(3)	51(4)	6(3)	3(3)	4(3)
C31	31(3)	32(3)	50(4)	-10(3)	4(3)	1(3)
C32	36(4)	33(3)	24(3)	-5(3)	1(3)	-3(3)
C33	37(4)	34(4)	53(4)	-1(3)	9(3)	4(3)
C34	36(3)	42(4)	35(3)	2(3)	14(3)	-2(3)
C35	54(4)	49(4)	37(4)	-3(3)	2(3)	11(3)
Se2	16.9(3)	22.8(3)	28.1(3)	-2.5(2)	7.2(2)	-3.9(2)
Se3	24.8(3)	24.7(3)	23.6(3)	3.4(3)	-7.2(2)	-2.2(3)
Se4	22.4(3)	29.5(4)	25.5(3)	8.5(3)	-4.1(2)	-3.9(3)
C11	30(4)	21(3)	22(4)	-2(3)	11(3)	-3(3)
C12	36(4)	23(3)	21(3)	-4(2)	15(3)	-3(3)
C13	24(3)	26(3)	31(3)	-9(3)	18(3)	-15(3)
C17	40(4)	26(3)	19(3)	5(2)	-5(3)	11(3)
C18	38(3)	21(3)	16(3)	2(2)	5(3)	2(3)
C19	26(3)	24(3)	15(3)	2(3)	10(2)	5(2)

C27	39(5)	60(5)	17(4)	-4(4)	1(3)	10(4)
C28	15(3)	47(4)	40(4)	5(3)	6(3)	-2(3)
C29	15(3)	29(4)	39(5)	0(3)	0(3)	3(3)
Se2A	16.9(3)	22.8(3)	28.1(3)	-2.5(2)	7.2(2)	-3.9(2)
Se3A	24.8(3)	24.7(3)	23.6(3)	3.4(3)	-7.2(2)	-2.2(3)
Se4A	22.4(3)	29.5(4)	25.5(3)	8.5(3)	-4.1(2)	-3.9(3)
C11A	30(4)	21(3)	22(4)	-2(3)	11(3)	-3(3)
C12A	36(4)	23(3)	21(3)	-4(2)	15(3)	-3(3)
C13A	24(3)	26(3)	31(3)	-9(3)	18(3)	-15(3)
C17A	40(4)	26(3)	19(3)	5(2)	-5(3)	11(3)
C18A	38(3)	21(3)	16(3)	2(2)	5(3)	2(3)
C19A	26(3)	24(3)	15(3)	2(3)	10(2)	5(2)
C27A	39(5)	60(5)	17(4)	-4(4)	1(3)	10(4)
C28A	15(3)	47(4)	40(4)	5(3)	6(3)	-2(3)
C29A	15(3)	29(4)	39(5)	0(3)	0(3)	3(3)

Table S4 Bond Lengths for Dye 14.

**Atom**	**Atom**	**Length/Å**		**Atom**	**Atom**	**Length/Å**
Se1	C1	1.843(5)		C20	C19	1.374(8)
Se1	C4	1.867(5)		C20	Se3A	1.742(15)
S1	C5	1.740(5)		C20	C19A	1.37(2)
S1	C9	1.739(5)		C21	C22	1.347(6)
S2	C21	1.738(5)		C22	C23	1.433(6)
S2	C25	1.741(5)		C23	C24	1.427(6)
P1	F1	1.590(4)		C24	C25	1.350(6)
P1	F2	1.584(4)		C25	C26	1.456(7)
P1	F3	1.586(4)		C26	Se4	1.852(5)
P1	F4	1.620(4)		C26	C27	1.387(9)
P1	F5	1.570(4)		C26	Se4A	1.762(10)
P1	F6	1.566(4)		C26	C27A	1.39(2)
N1	C30	1.126(7)		C30	C31	1.457(8)
N2	C32	1.127(7)		C32	C33	1.455(8)
N3	C34	1.137(8)		C34	C35	1.474(9)
C1	C2	1.342(7)		Se2	C13	1.849(5)
C2	C3	1.429(7)		Se3	C17	1.853(6)

C3	C4	1.387(7)		Se4	C29	1.854(6)
C4	C5	1.458(6)		C11	C12	1.416(9)
C5	C6	1.346(6)		C12	C13	1.365(8)
C6	C7	1.420(6)		C17	C18	1.350(8)
C7	C8	1.434(6)		C18	C19	1.401(8)
C7	C14	1.407(6)		C27	C28	1.407(9)
C8	C9	1.348(6)		C28	C29	1.344(9)
C9	C10	1.462(6)		Se2A	C13A	1.85(2)
C10	Se2	1.865(5)		Se3A	C17A	1.85(2)
C10	C11	1.369(8)		Se4A	C29A	1.85(2)
C10	Se2A	1.785(14)		C11A	C12A	1.41(2)
C10	C11A	1.36(2)		C12A	C13A	1.36(2)
C14	C15	1.388(6)		C17A	C18A	1.35(2)
C15	C16	1.391(6)		C18A	C19A	1.40(2)
C16	C23	1.410(6)		C27A	C28A	1.40(2)
C20	C21	1.458(6)		C28A	C29A	1.34(2)
C20	Se3	1.855(5)				

Table S5 Bond Angles for Dye 14.								
**Atom**	**Atom**	**Atom**	**Angle/°**		**Atom**	**Atom**	**Atom**	**Angle/°**
C1	Se1	C4	87.3(2)		C19	C20	Se3	110.2(4)
C9	S1	C5	103.0(2)		C19A	C20	C21	124(5)
C21	S2	C25	103.2(2)		C19A	C20	Se3A	109(5)
F1	P1	F4	89.5(2)		C20	C21	S2	113.6(4)
F2	P1	F1	90.3(2)		C22	C21	S2	122.4(4)
F2	P1	F3	91.1(2)		C22	C21	C20	124.0(4)
F2	P1	F4	179.2(3)		C21	C22	C23	126.4(4)
F3	P1	F1	178.4(2)		C16	C23	C22	117.6(4)
F3	P1	F4	89.0(2)		C16	C23	C24	123.1(4)
F5	P1	F1	90.4(2)		C24	C23	C22	119.3(4)
F5	P1	F2	91.2(2)		C25	C24	C23	125.3(4)
F5	P1	F3	88.9(2)		C24	C25	S2	123.4(4)
F5	P1	F4	88.1(2)		C24	C25	C26	123.5(4)
F6	P1	F1	90.4(2)		C26	C25	S2	113.2(3)
F6	P1	F2	92.4(2)		C25	C26	Se4	121.8(3)
F6	P1	F3	90.2(2)		C25	C26	Se4A	124.5(5)
F6	P1	F4	88.3(2)		C27	C26	C25	127.8(5)
F6	P1	F5	176.3(2)		C27	C26	Se4	110.4(4)
C2	C1	Se1	112.7(4)		C27A	C26	C25	128(4)

C1	C2	C3	115.4(5)		C27A	C26	Se4A	108(4)
C4	C3	C2	113.7(5)		N1	C30	C31	177.7(7)
C3	C4	Se1	111.0(4)		N2	C32	C33	178.1(7)
C3	C4	C5	127.2(4)		N3	C34	C35	179.2(8)
C5	C4	Se1	121.8(3)		C13	Se2	C10	86.9(2)
C4	C5	S1	113.8(3)		C17	Se3	C20	87.2(2)
C6	C5	S1	122.9(4)		C26	Se4	C29	87.8(3)
C6	C5	C4	123.3(4)		C10	C11	C12	114.4(7)
C5	C6	C7	126.4(4)		C13	C12	C11	114.8(5)
C6	C7	C8	118.7(4)		C12	C13	Se2	112.2(4)
C14	C7	C6	123.2(4)		C18	C17	Se3	112.2(4)
C14	C7	C8	118.1(4)		C17	C18	C19	114.2(5)
C9	C8	C7	126.3(4)		C20	C19	C18	116.0(5)
C8	C9	S1	122.6(4)		C26	C27	C28	114.5(6)
C8	C9	C10	123.5(4)		C29	C28	C27	115.9(7)
C10	C9	S1	113.9(3)		C28	C29	Se4	111.3(5)
C9	C10	Se2	121.4(4)		C10	Se2A	C13A	87(3)
C9	C10	Se2A	130.6(7)		C20	Se3A	C17A	88(5)
C11	C10	C9	126.9(5)		C26	Se4A	C29A	90(3)
C11	C10	Se2	111.7(4)		C10	C11A	C12A	112(2)
C11A	C10	C9	114(4)		C13A	C12A	C11A	116(2)
C11A	C10	Se2A	115(4)		C12A	C13A	Se2A	110(4)
C15	C14	C7	124.6(4)		C18A	C17A	Se3A	114(10)
C14	C15	C16	123.1(4)		C17A	C18A	C19A	107(10)
C15	C16	C23	125.1(5)		C20	C19A	C18A	121(10)
C21	C20	Se3	121.9(4)		C26	C27A	C28A	121(8)
C21	C20	Se3A	127.0(7)		C29A	C28A	C27A	109(10)
C19	C20	C21	127.9(5)		C28A	C29A	Se4A	113(7)

Table S6 Torsion Angles for Dye 14.										
**A**	**B**	**C**	**D**	**Angle/°**		**A**	**B**	**C**	**D**	**Angle/°**
Se1	C1	C2	C3	-0.7(6)		C24	C25	C26	C27	-175.9(6)
Se1	C4	C5	S1	-177.6(2)		C24	C25	C26	Se4A	168.8(6)
Se1	C4	C5	C6	0.4(7)		C24	C25	C26	C27A	-5(3)
S1	C5	C6	C7	0.4(7)		C25	S2	C21	C20	177.7(3)
S1	C9	C10	Se2	176.5(2)		C25	S2	C21	C22	-2.1(5)
S1	C9	C10	C11	-2.9(7)		C25	C26	Se4	C29	-180.0(4)
S1	C9	C10	Se2A	2.2(11)		C25	C26	C27	C28	-178.4(6)
S1	C9	C10	C11A	174(3)		C25	C26	Se4A	C29A	-175(2)
S2	C21	C22	C23	-0.2(7)		C25	C26	C27A	C28A	176(3)
S2	C25	C26	Se4	-177.2(3)		C26	Se4	C29	C28	0.3(5)
S2	C25	C26	C27	6.0(8)		C26	C27	C28	C29	-4.5(10)
S2	C25	C26	Se4A	-9.4(7)		C26	Se4A	C29A	C28A	-2(3)
S2	C25	C26	C27A	177(3)		C26	C27A	C28A	C29A	-2(4)
C1	Se1	C4	C3	0.2(4)		Se2	C10	C11	C12	0.7(7)
C1	Se1	C4	C5	179.8(4)		Se2	C10	Se2A	C13A	-4(3)
C1	C2	C3	C4	0.9(7)		Se2	C10	C11A	C12A	23(20)
C2	C3	C4	Se1	-0.7(6)		Se3	C20	C21	S2	173.5(2)
C2	C3	C4	C5	179.8(5)		Se3	C20	C21	C22	-6.7(7)
C3	C4	C5	S1	1.8(7)		Se3	C20	C19	C18	1.6(6)
C3	C4	C5	C6	179.9(5)		Se3	C20	Se3A	C17A	12(3)
C4	Se1	C1	C2	0.3(4)		Se3	C20	C19A	C18A	-94(28)
C4	C5	C6	C7	-177.5(4)		Se3	C17	C18	C19	0.3(7)
C5	S1	C9	C8	1.8(5)		Se4	C26	C27	C28	4.5(8)
C5	S1	C9	C10	-177.6(3)		Se4	C26	Se4A	C29A	-6(2)
C5	C6	C7	C8	-0.1(7)		Se4	C26	C27A	C28A	134(22)
C5	C6	C7	C14	179.6(5)		C11	C10	Se2	C13	-0.3(5)
C6	C7	C8	C9	1.0(7)		C11	C10	Se2A	C13A	-141(11)
C6	C7	C14	C15	-2.1(7)		C11	C10	C11A	C12A	5(2)

C7	C8	C9	S1	-2.0(7)		C11	C12	C13	Se2	0.6(7)
C7	C8	C9	C10	177.3(4)		C17	C18	C19	C20	-1.3(8)
C7	C14	C15	C16	178.6(4)		C19	C20	C21	S2	-7.8(7)
C8	C7	C14	C15	177.7(4)		C19	C20	C21	C22	172.0(5)
C8	C9	C10	Se2	-2.9(6)		C19	C20	Se3	C17	-1.1(4)
C8	C9	C10	C11	177.7(6)		C19	C20	Se3A	C17A	-84(4)
C8	C9	C10	Se2A	-177.1(10)		C19	C20	C19A	C18A	14(3)
C8	C9	C10	C11A	-5(3)		C27	C26	Se4	C29	-2.7(5)
C9	S1	C5	C4	177.0(3)		C27	C26	Se4A	C29A	76(3)
C9	S1	C5	C6	-1.1(5)		C27	C26	C27A	C28A	-12(2)
C9	C10	Se2	C13	-179.8(4)		C27	C28	C29	Se4	2.2(8)
C9	C10	C11	C12	-179.8(5)		Se2A	C10	Se2	C13	-4.4(8)
C9	C10	Se2A	C13A	170(3)		Se2A	C10	C11	C12	46(10)
C9	C10	C11A	C12A	-172(2)		Se2A	C10	C11A	C12A	1(2)
C10	Se2	C13	C12	-0.2(4)		Se3A	C20	C21	S2	10.1(10)
C10	C11	C12	C13	-0.9(8)		Se3A	C20	C21	C22	-170.2(9)
C10	Se2A	C13A	C12A	2(4)		Se3A	C20	Se3	C17	-16.1(8)
C10	C11A	C12A	C13A	1(3)		Se3A	C20	C19	C18	91(3)
C14	C7	C8	C9	-178.7(5)		Se3A	C20	C19A	C18A	-1(3)
C14	C15	C16	C23	179.5(4)		Se3A	C17A	C18A	C19A	-1(3)
C15	C16	C23	C22	179.1(4)		Se4A	C26	Se4	C29	10.9(5)
C15	C16	C23	C24	-0.9(7)		Se4A	C26	C27	C28	-99(3)
C16	C23	C24	C25	177.5(5)		Se4A	C26	C27A	C28A	1(2)
C20	C21	C22	C23	-179.9(4)		C11A	C10	Se2	C13	-163(20)
C20	Se3	C17	C18	0.5(5)		C11A	C10	C11	C12	3(3)
C20	Se3A	C17A	C18A	0.1(15)		C11A	C10	Se2A	C13A	-2(3)
C21	S2	C25	C24	2.4(5)		C11A	C12A	C13A	Se2A	-2(4)
C21	S2	C25	C26	-179.5(3)		C17A	C18A	C19A	C20	1(4)
C21	C20	Se3	C17	177.8(4)		C19A	C20	C21	S2	-174(4)
C21	C20	C19	C18	-177.3(5)		C19A	C20	C21	C22	6(4)
C21	C20	Se3A	C17A	177(3)		C19A	C20	Se3	C17	75(28)
C21	C20	C19A	C18A	-178(3)		C19A	C20	C19	C18	-10(3)
C21	C22	C23	C16	-177.2(5)		C19A	C20	Se3A	C17A	0.6(14)
C21	C22	C23	C24	2.8(7)		C27A	C26	Se4	C29	-38(23)
C22	C23	C24	C25	-2.5(7)		C27A	C26	C27	C28	9(2)
C23	C24	C25	S2	-0.3(7)		C27A	C26	Se4A	C29A	0.4(13)
C23	C24	C25	C26	-178.3(5)		C27A	C28A	C29A	Se4A	2(4)
C24	C25	C26	Se4	0.9(7)						

Table S7 Atomic Occupancy for Dye 14.							
**Atom**	***Occupancy***		**Atom**	***Occupancy***		**Atom**	***Occupancy***
Se2	0.9565(17)		Se3	0.9621(17)		Se4	0.9255(18)
C11	0.9565(17)		H11	0.9565(17)		C12	0.9565(17)
H12	0.9565(17)		C13	0.9565(17)		H13	0.9565(17)
C17	0.9621(17)		H17	0.9621(17)		C18	0.9621(17)
H18	0.9621(17)		C19	0.9621(17)		H19	0.9621(17)
C27	0.9255(18)		H27	0.9255(18)		C28	0.9255(18)
H28	0.9255(18)		C29	0.9255(18)		H29	0.9255(18)
Se2A	0.0435(17)		Se3A	0.0379(17)		Se4A	0.0745(18)
C11A	0.0435(17)		H11A	0.0435(17)		C12A	0.0435(17)
H12A	0.0435(17)		C13A	0.0435(17)		H13A	0.0435(17)
C17A	0.0379(17)		H17A	0.0379(17)		C18A	0.0379(17)
H18A	0.0379(17)		C19A	0.0379(17)		H19A	0.0379(17)
C27A	0.0745(18)		H27A	0.0745(18)		C28A	0.0745(18)
H28A	0.0745(18)		C29A	0.0745(18)		H29A	0.0745(18)

(1) Dolomanov, O. V.; Bourhis, L. J.; Gildea, R. J.; Howard, J. A. K.; Puschmann, H. *J. Appl. Crystallogr.* **2009**, *42*, 339.

(2) Sheldrick, G. M. *Acta Cryst.* **2008**, *A64*, 112.
